# A Nicotinamide Phosphoribosyltransferase Inhibitor, FK866, Suppresses the Growth of Anaplastic Meningiomas and Inhibits Immune Checkpoint Expression by Regulating STAT1

**DOI:** 10.3389/fonc.2022.836257

**Published:** 2022-04-20

**Authors:** Yuxuan Deng, Boyi Hu, Yazhou Miao, Jing Wang, Shaodong Zhang, Hong Wan, Zhen Wu, Yifan Lv, Jie Feng, Nan Ji, Deric Park, Shuyu Hao

**Affiliations:** ^1^ Department of Neurosurgery, Beijing Tiantan Hospital, Capital Medical University, Beijing, China; ^2^ Beijing Neurosurgical Institute, Capital Medical University, Beijing, China; ^3^ Department of Neurology, University of Chicago Medical Center, Chicago, IL, United States

**Keywords:** anaplastic meningioma, NAMPT, NAMPT inhibitor, immune checkpoint, cell cycle, proteomics

## Abstract

Anaplastic meningioma is classified as a World Health Organization (WHO) grade III tumor and shows a strong tendency to recur. Although the incidence of anaplastic meningioma is low, the high rate of recurrence and death still makes treatment a challenge. A proteomics analysis was performed to investigate the differentially expressed proteins between anaplastic meningiomas and fibrous meningiomas by micro-LC-MS/MS. The key metabolic enzyme nicotinamide phosphoribosyltransferase (NAMPT) showed upregulated expression in anaplastic meningiomas. However, targeting NAMPT to treat anaplastic meningiomas has not been reported. *In vitro*, NAMPT inhibitor -FK866 reduced the viability of anaplastic meningiomas by inducing cell cycle arrest at the G2/M phase. Intriguingly, the NAMPT inhibitor -FK866 decreased the protein expression of immune checkpoints PD-L1 and B7-H3 by down-regulating the STAT1 and p-STAT1 expression *in vitro*. Furthermore, FK866 suppressed the growth of anaplastic meningiomas in an *in vivo* xenograft model. The expression of Ki-67 and immune checkpoint proteins (PD-L1 and B7-H3) showed significant differences between the group treated with FK866 and the control group treated with DMSO. In conclusion, the expression of NAMPT, which plays a crucial role in energy metabolism, was upregulated in anaplastic meningiomas. The NAMPT inhibitor -FK866 significantly suppressed the growth of anaplastic meningiomas *in vitro* and *in vivo*. More strikingly, FK866 potently inhibited immune checkpoint protein (PD-L1 and B7-H3) expression by regulating STAT1 *in vitro* and *in vivo*. Our results demonstrated that NAMPT inhibitors could potentially be an effective treatment method for patients suffering from anaplastic meningiomas.

## Introduction

Anaplastic meningioma is classified as a World Health Organization (WHO) grade III tumor. The incidence of anaplastic meningioma is low, but the high rate of recurrence and related deaths make treatment a challenge. The 5-year overall survival rate for patients with anaplastic meningioma is 35%–61% (1–3). The treatment strategy is complete tumor resection (4). Radiotherapy approaches are also used for the treatment of anaplastic meningiomas when surgery alone is insufficient (5). However, there is no known effective medical therapy for anaplastic meningiomas (6). Therefore, it is necessary to find a novel therapy for patients with anaplastic meningiomas.

Nicotinamide phosphoribosyltransferase (NAMPT) is an enzyme involved in nicotinamide adenine dinucleotide (NAD) biosynthesis (7). NAD is an important coenzyme for redox reactions, making it central to energy metabolism. Moreover, NAD is a cofactor or substrate for hundreds of enzymes, and it can directly and indirectly influence many key cellular functions, including metabolic pathways, DNA repair, chromatin remodeling, cellular senescence and immune function. Because of the Warburg effect (8), cancer cells need to maintain a redox state and synthesize building blocks to support tumor proliferation and progression, which means they need high levels of NAD. Previous studies have shown that NAD is synthesized from tryptophan, nicotinic acid (NA) and nicotinamide (NM) through three major pathways: the *de novo*, Preiss-Handler, and salvage pathways (9–11) (**Figure 3**). NAMPT is the key rate-limiting enzyme in the salvage pathway.

High levels of NAMPT expression have been observed in many studies (12–15). NAMPT can facilitate tumor initiation and progression and it can induce cancer stem cell-like properties in colon cancer and glioma (15, 16). In breast cancer, NAMPT can facilitate tumor cell proliferation and invasiveness (17). It is also important for prostate cell growth and survival (18). Recent studies have also shown that NAMPT serves as a promoter of an immunosuppressive environment. Travelli et al. found that NAMPT can facilitate the mobilization of immature myeloid-derived suppressor cells (MDSCs), and this mobilization can be suppressed by NAMPT inhibitors (19). Audrito V, et al. found that NAMPT plays an important role in macrophage differentiation to the M2 phenotype and polarization in tumorigenesis-associated macrophages in leukemia (20). In general, NAMPT can promote the progression of tumors and regulate the tumor immune microenvironment.

Given the high turnover of NAD in cancer cells and the fact that NAMPT is the rate-limiting enzyme in the salvage pathway, inhibitors of NAMPT were first reported as possible anticancer agents by Hasmann et al. in 2003 (21). As a kind of small-molecule NAMPT inhibitor, FK866 has shown anticancer activity in several tumor models by depleting NAD levels (21–23). Other studies have shown that FK866 can inhibit the growth of tumors and induce the apoptosis of cancer cells (24, 25). However, the specific anticancer mechanism of FK866 is still unknown in anaplastic meningiomas.

Previous studies have shown that the inhibition of NAMPT expression can dramatically reduce the activation of STAT1 (26). STAT1 performs various important biological functions in normal cells, such as promoting cell death, inhibiting cell growth, stimulating the immune system, and regulating cell differentiation. In tumors, STAT1 is often considered to be a promotor of antitumor activity, but STAT1 induction has also been implicated in cancer progression. For example, STAT1 plays a critical role in mediating IFN-induced PD-L1 transcription in cancer cells (27), blocking T cell activation and causing tumor immune escape.

In this study, we assessed whether NAMPT plays a critical role during the tumorigenesis of anaplastic meningiomas and investigated the potential mechanism by which FK866 suppresses the growth of tumors. We also aimed to determine whether NAMPT regulates the immune checkpoint in anaplastic meningiomas by regulating STAT1.

## Methods

### Patients and Specimens

Six patients were diagnosed with fibrous meningiomas and four patients were diagnosed with anaplastic meningiomas. The diagnosis was based on pathological examination. All patients underwent surgery at Beijing Tiantan Hospital, Capital Medical University. Fresh tumor tissue samples from these patients were frozen at − 80°C in isopentane and stored in liquid nitrogen. All tumor tissue samples were used for proteomic analysis. All nine of tumor tissues were used for Western blot analysis and immunohistochemical staining. This study was approved by the Ethics Committees of Beijing Tiantan Hospital (KY2021-158-01). Informed consent was obtained from all enrolled subjects, and the study was performed in full compliance with all principles of the Declaration of Helsinki.

### Protein Preparation and Nano-Liquid Chromatography With Tandem Mass Spectroscopy (Micro-LC-MS/MS) Analysis

**Figure 1:**
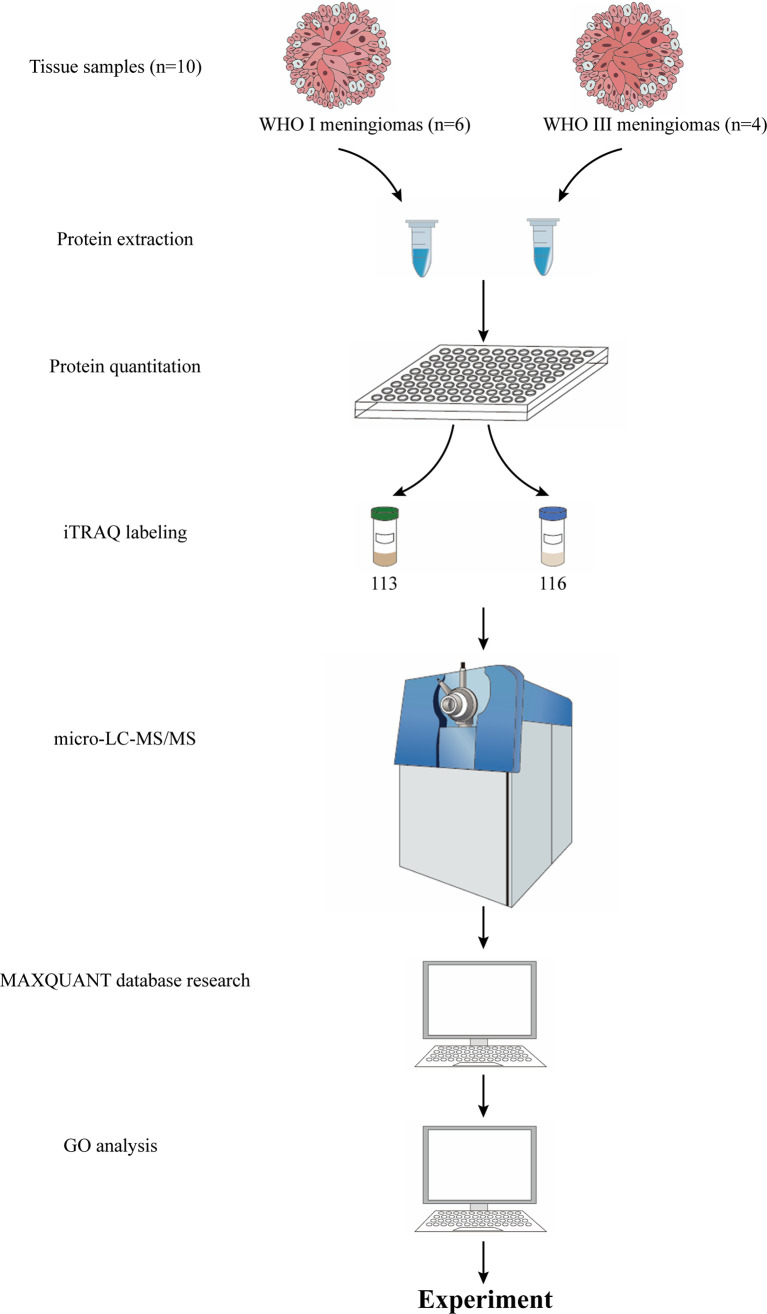
The workflow of proteomic strategy.

The workflow of protein preparation and proteomics analysis is shown in **Figure 1**. The protein from tissue samples was extracted with lysis buffer (4% SDS and 100 mM HEPES, pH 7.6). The homogenate was sonicated for 10 min on ice. After centrifugation at 25,000 g for 30 min at 4°C, the supernatant was collected and stored at − 80°C. The total protein concentration was measured with a bicinchoninic acid (BCA) kit (23227, Pierce, Rockford, IL, USA).

Equal protein samples from each of six fibrous meningiomas or four anaplastic meningioma tissues were combined into a single pool, as shown in **Figure 1**. A mass of 200 mg of each pooled sample was reduced using reducing reagent at 60°C for 1h and alkylated with cysteine blocking reagent at room temperature for 10 min as described in the iTRAQ protocol (Applied Biosystems). Trypsin was added to the sample at a mass ratio of 1:50 (enzyme:protein) and incubated at 37°C overnight. The digested samples were labeled with 113 and 116 iTRAQ tags, respectively, as shown in **Figure 1**, according to the manufacturer’s protocol (AB Sciex). The tagged peptides were dried *via* vacuum centrifugation and combined in one tube. The pooled sample was separated on apoly-LC SCX column (4.6 × 250 mm, 5 μm, 100 Å) using an Nexera XR instrument (LC-20ADXR, Shimadzu), and the labeled peptides were detected by ultraviolet radiation using SPD-20A (Shimadzu, Japan). In this study, a total of 55 fractions were collected, dried by speed vacuum centrifugation, and combined into 12 fractions according to the SCX chromatogram. Each fraction was injected onto a desalting column (10 × 0.3 mm, 5 μm-C18, 120 Å) and separated on an analytical column (0.3 × 150 mm, 3μmC18, 120 Å) using an Eksigent microLC instrument (Eksigent, Dublin, CA, USA). The samples separated *via* capillary high-performance liquid chromatography were subsequently analyzed using a Triple TOF 5600+ system (AbSciex, USA).

Protein identification and proteome annotation were performed using the ProteinPilot™ software package 5.0 (Applied Biosystems) and searched against the SwissProt database (March 2020). The following search parameters were utilized to analyze the MS/MS data: trypsin as the digestion enzyme with a maximum of two missed cleavages allowed; fixed modifications of carbamidomethyl (C) and iTRAQPlex (K and N-terminus); variable modifications of oxidation (M); peptide mass tolerance of ±20 ppm; fragment mass tolerance of ±0.1 Da; and peptide FDR ≤ 0.01.

The mass spectrometry proteomics data have been deposited to the ProteomeXchange Consortium (http://proteomecentral.proteomexchange.org) *via* the iProX partner repository (28) with the dataset identifier PXD032342.

### Bioinformatics Analysis

The differentially expressed proteins were analyzed *via* enrichment analyses using Gene Ontology (GO) (available at www.geneontology.org) for the identification of associated biological processes and molecular functions.

### Cell Culture and Viability Measurement

The anaplastic meningioma cell line (CRL-3370) was obtained from the American Type Culture Collection. The cell culture medium consisted of DMEM supplemented with 10% fetal bovine serum (FBS) (Gibco, USA). The cell line was maintained at 37 °C in a humidified incubator with 5% CO_2_.

Cell viability was determined using Cell Counting Kit-8 (CCK-8) (Biosharp, China, BS350B). Briefly, 5mg FK866 (Selleck, USA, S2799) was dissolved in 1.2771 ml dimethyl sulfoxide (DMSO) (Sigma, USA, D2650), then 10 mM FK866 was obtained. Then 10 mM FK866 was diluted with DMSO to obtain 10 μM FK866. After incubation with DMSO or different concentration of FK866 (25μM, 12.5μM, 2.5μM, 0.01μM, 0.005μM) for 24h, 48h or 72 h, 10 µl of CCK-8 reagent was added to each well, and the plates were further incubated in an incubator for 2h. Subsequently, the absorbance was measured at 450 nm by a microplate reader (Biotex, Synergy H1, USA). The cell viability (%) was calculated as follows: (average OD of treated groups at 24, 48 or 72 h/average OD of untreated groups at the same time point) x 100%.

### Colony Formation Assay

The indicated cells were digested and resuspended and counted under a microscope. The cells were cultured in 6-cm plates at a density of 500 cells per well. The cells were cultured under normal culture conditions with DMSO or FK866 for 7 days. The supernatant was removed, the cells were fixed with paraformaldehyde, and the cells were dyed with purple crystal. Then, the plates were washed with PBS twice. The whole field of view was photographed and counted. ImageJ (V1.48, NIH, Bethesda, MD, USA) was used for counting and measurement of colony mean size as described earlier (29). In brief, after converting the image to 8-bit format, the threshold was adjusted to exclude the dazzle. Then, colonies were counted and measured using the “Analyze Particles” function.

### Western Blot Analysis

Tumor cells were lysed using RIPA buffer with a protease inhibitor cocktail (Millipore Sigma). Lysates were separated by SDS-PAGE (Bio-Rad) and transferred to PVDF membranes. The membranes were probed for NAMPT (Abcam, ab236874), STAT1 (CST, 14994), phosphorylated(p)-STAT1 (CST, 9167), PD-L1 (CST, 13684), B7-H3 (CST, 14058) and β-actin (Abcam, ab20272).

### Immunohistochemical Staining

Immunohistochemical staining was performed on consecutive sections from xenograft tumors removed from nude mice treated with vehicle controls and FK866 (5 mg/kg by intraperitoneal (i.p.) injection once per day). The tumor samples were fixed in 10% buffered formalin and embedded in paraffin. The sections cut from paraffin embedded blocks were deparaffinized with xylene and ethanol, and then immersed for 40 min in 10 mM citric acid buffer (pH 6.0) or EDTA antigen retrieval solution (pH 9.0). The activity of endogenous peroxides was quenched for 20 min in 3% H_2_O_2_ in methanol. After rinsing in PBS, the sections were incubated at 4°C with anti-Ki-67 (Abcam, ab16667), anti-PD-L1 (CST, 13684) and anti-B7-H3 (CST, 14058) antibodies overnight. Then, a brown immunostain was developed using 3,3-diaminobenzidine tetrahydrochloride and hydrogen peroxidase, and counterstained with hematoxylin. The sections were then dehydrated, cleared with xylene and scanned with a digital slice scanning system (Leica, AT2, USA).

### Reverse Transcription-Quantitative Polymerase Chain Reaction (RT-qPCR)

Total RNA was isolated from IOMM cell line using QIAzol Lysis Reagent (Qiagen Sciences, Maryland, USA). cDNA was synthesized using a High Capacity cDNA Reverse Transcription Kit (Applied Biosystems, CA, USA). The cDNA was subsequently analyzed using QuantStudio 5 (Applied Biosystems, CA, USA). The amplification program was as follows: initial denaturation step at 95°C for 30 s, followed by 40 cycles at 95°C for 15 s and 60°C for 60 s. The expression of each specific gene was calculated relative to the expression of the internal reference gene GAPDH using the 2–ΔΔCt method. The primer sequences are shown in **Supplementary Table 1**. All assays were performed in triplicate.

### Staining With 5-ethynyl-2′-deoxyuridine (Edu)

An EdU (RiboBio, China, C10310-1) assay was used to determine the cell proliferation rate. Cells were seeded in 96-well plates (1×10^3^ cells/well) and cultured with DMEM (10% FBS) for 48 h. Then, 100 µl of EdU solution (50 µM) was added to each well and incubated for 2 h at 37°C. Then, the cells were fixed with 4% formaldehyde for 30 min and permeabilized with 0.5% Triton X-100 for 10 min at room temperature. Next the cells were washed with PBS for 5 min and 1X ApolloR reaction cocktail was added to each well (100 µl/well). Thirty minutes later, the cells were incubated with 100 µl Hoechst 33342 for 30 min. Finally, the cells were observed with an inverted microscope (Zeiss, Axio Observer. ZI, USA).

### Cell Cycle Assay

The Cell Cycle and Apoptosis Analysis Kit (Biosharp, China, BL114A) was used to detect cell cycle of IOMM cells treated with FK866 or DMSO. The cells were washed with cold PBS, fixed in 70% ethanol, and stored at 4°C for subsequent cell cycle analysis. The fixed cells were washed with PBS once and then re-suspended in 1 mL of PI staining reagent (50 mg/ml propidium iodide and 1 mg/ml RNAse in 1 ml of sodium citrate buffer, pH 7.4). The samples were incubated in the dark for 30 min before cell cycle analysis. The distribution of cells in the cell cycle was measured by an Amnis imaging flow cytometer (ImageStreamX MarkII), and quantitation of cell cycle distribution was performed using FlowJo 10.6.2 Software. The percentages of cells in the G1, S and G2 phases were calculated.

### Xenograft Experiments

To construct the subcutaneous tumor xenograft mouse model, 2×10^6^ IOMM cells were suspended in 100 μL of solution (PBS) and injected subcutaneously into the right dorsal flank of 6–8-week-old female nude mice. Tumors were allowed to establish until the tumor size was measurable, and then the mice were randomly assigned to be treated for two weeks with vehicle controls or FK866 (5 mg/kg i.p. once per day). The dose of the compound was determined based on previous studies (30–32). FK866 was suspended in 45% propylene glycol + 5% Tween 80 + double distilled H_2_O (ddH_2_O). Tumor size was measured twice per week by two investigators as follows: tumor size (mm^3^) = [d^2^ x D]/2, where d is the shortest diameter and D is the largest diameter. After two weeks of treatments, tumor samples were surgically removed and the tumor weights were measured as a surrogate for tumor burden.

### Statistical Analysis

The data obtained from the above experiments were statistically analyzed with SPSS 22.0 software. The measurement data are expressed by as the mean ± standard deviation (SD). T tests were performed to compare the differences between two groups. One-way ANOVA was performed to compare the differences between the three groups. Each experiment was repeated at least 3 times. P < 0.05 was considered to be statistically significant.

## Results

### NAMPT Was Upregulated in Human Anaplastic Meningioma Tissues

In the present study, we explored differentially expressed proteins between fibrous meningioma and anaplastic meningioma tissues by performing a proteomic analysis. In total, 429 differentially expressed proteins were identified. Of these proteins, 352 proteins were upregulated and 77 proteins were downregulated in anaplastic meningioma tissues (with both a P value and false discovery rate (FDR) both < 0.05, with fold change > 1.5 or < 0.67) (**Supplementary Material 2**).

**Figure 2:**
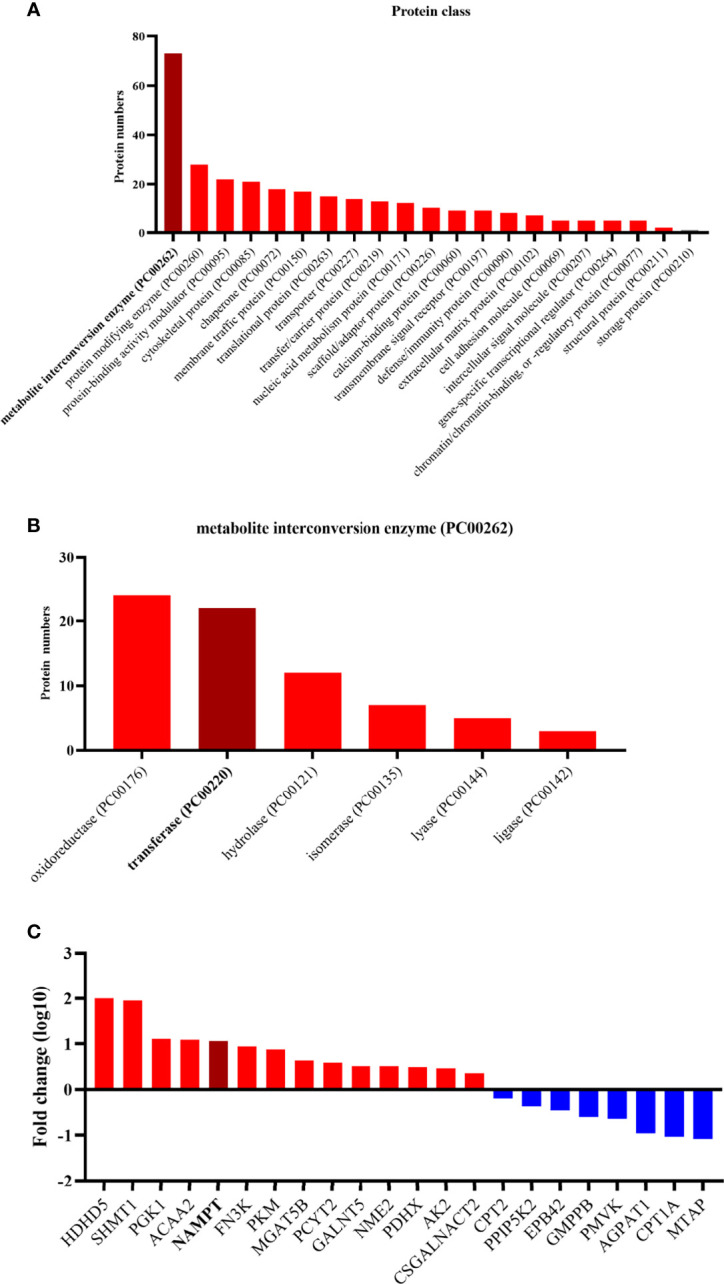
**(A)** The twenty enriched biological processes according to GO analysis. Most of differentially expressed proteins are metabolite interconversion enzymes (PC00262). **(B)** The different functions of the metabolite interconversion enzymes (PC00262). Oxidoreductase (PC00176) and transferase (PC00220) enzymes accounts for more than half of the metabolic interconversion enzymes. **(C)** Diagram listing the transferases related to the differentially expressed proteins. The y-axis represents the fold change, and the x-axis lists the names of transferases. Red represents a high expression level in anaplastic meningiomas, while blue represents a low expression level.

Based on the protein class of Protein Analysis Through Evolutionary Relationships (PANTHER) Classification System in the Gene Oncology (GO) database (www.geneontology.org) (33), all of these differential proteins were classified (**Figure 2**). A large portion of the differentially expressed proteins was involved with metabolite interconversion enzymes (PC00262). A third of the metabolite interconversion enzymes were transferases (PC00220). Of these transferases, 14 proteins were upregulated and 8 proteins were downregulated in anaplastic meningiomas. The expression of NAMPT was obviously different between fibrous meningiomas and anaplastic meningiomas.

**Figure 3:**
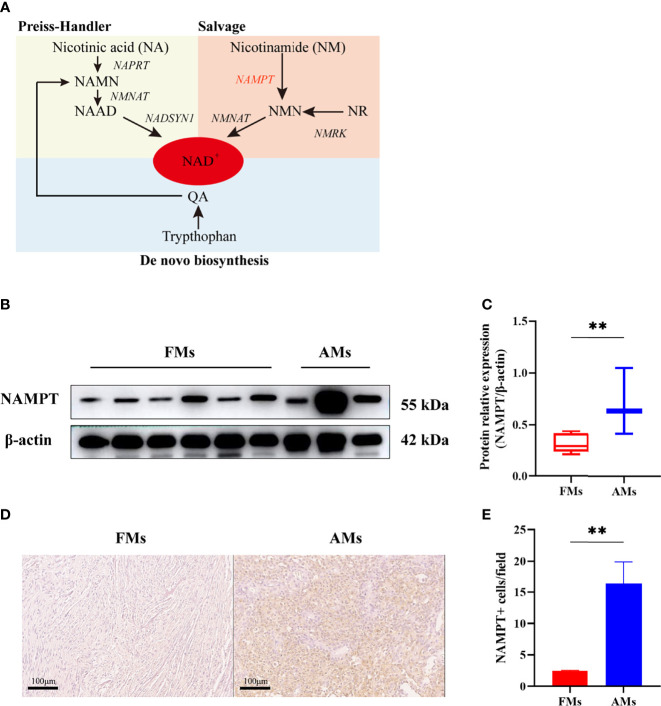
**(A)** NAD is synthesized from tryptophan, nicotinic acid (NA) and nicotinamide (NM) through *de novo*, Preiss-Handler, and salvage pathways. **(B, C)** The relative expression of NAMPT (NAMPT/β-actin) in fibrous meningioma samples (n=6) and anaplastic meningiomas samples (n=3) was detected by Western blotting. **(D, E)** The expression of NAMPT in fibrous meningioma samples and anaplastic meningiomas samples was detected by immunohistochemical staining. FMs: fibrous meningiomas AMs: anaplastic meningiomas. **p < 0.01.

To validate the differential expression of NAMPT between fibrous meningiomas and anaplastic meningiomas, we detected the protein level of NAMPT in fibrous meningioma samples (n=6) and anaplastic meningiomas samples (n=3) by Western blot analysis. There was a significant difference in NAMPT expression between fibrous meningiomas and anaplastic meningiomas (P=0.0229) (**Figures 3B, C**). The expression of NAMPT was significantly higher in the anaplastic meningioma tissues than in the fibrous meningioma tissues. We also detected the expression of NAMPT in tissues by immunohistochemical staining. There was a significant difference of NAMPT expression between fibrous meningioma tissues and anaplastic meningioma tissues (P=0.0024) (**Figures 3D, E**). The expression of NAMPT was significantly higher in anaplastic meningiomas than in fibrous meningioma tissues.

### The Inhibition of NAMPT by FK866 Suppressed the Proliferation of IOMM Cells *In Vitro*

**Figure 4:**
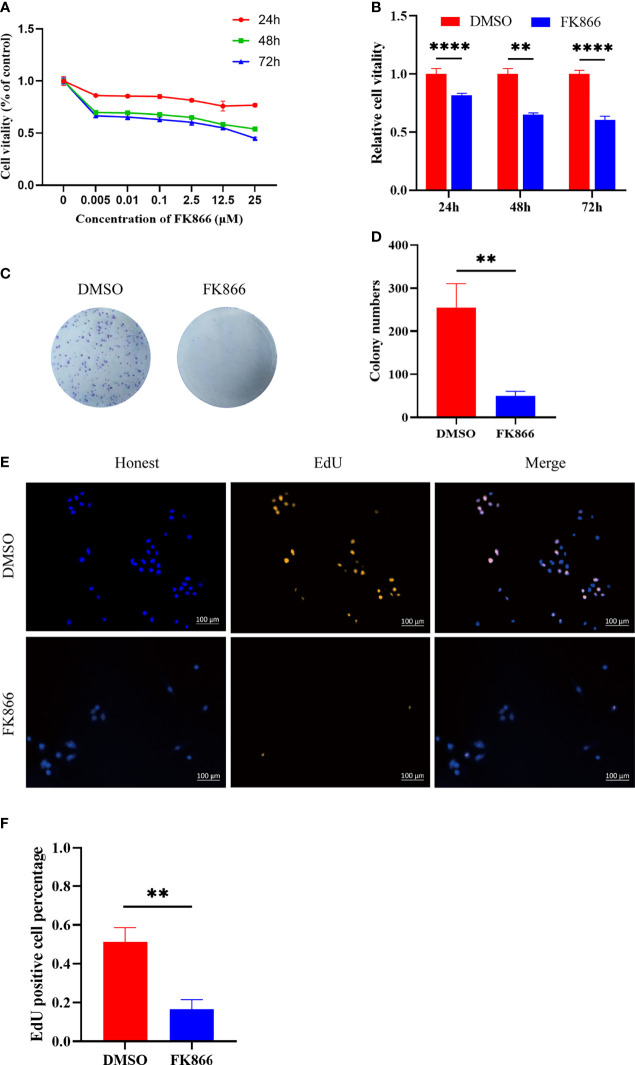
**(A, B)** Inhibitory effects of FK866 on IOMM cells as demonstrated using the CCK-8 assay. The cells were treated with various concentrations of FK866 (0–25 µM) for 24, 48 and 72 h. **(C, D)** The proliferation of IOMM cells treated with FK866 or DMSO was detected by colony formation assay. **(E, F)** Percentage of EdU positive cells among IOMM cells. **p < 0.01, ****p < 0.0001.

In this study, the inhibitory effect of FK866 on the viability of IOMM cells was investigated at different concentrations (0-25 μM) (**Figure 4A**). The viability of the IOMM cells was detected at different times (24, 48 and 72 h). A dose-dependent inhibition of cell growth was observed with increasing FK866 concentration at 24, 48 and 72 h. We also thought that a high concentration of FK866 induces toxicity in normal cells. We therefore chose the dose of 2.5 μM in subsequent *in vitro* studies to examine the effect of FK866 on IOMM cells. A significant difference was observed between the DMSO group and the FK866-treated groups at 2.5 μM after 48 h of incubation (p=0.0022) (**Figure 4B**). The effect of FK866 on IOMM proliferation was also detected by colony formation and EdU assays. The colony formation assays revealed that FK866 inhibited colony formation of IOMM cells (**Figure 4C**). Colony numbers were significantly different between the FK866‐treated group and DMSO-treated group (p=0.0032) (**Figure 4D**). The results of the EdU assay are shown in **Figures 4E, F**. The number of cells in the proliferative phase was significantly between the FK866‐treated group and the DMSO-treated group (p=0.0025). There were fewer cells in the proliferative phase in the FK866-treated group than in the DMSO-treated group. In summary, FK866 inhibited the proliferation of IOMM cells.

### The Inhibition of NAMPT Was Effective Against Cell Cycle Progression

**Figure 5:**
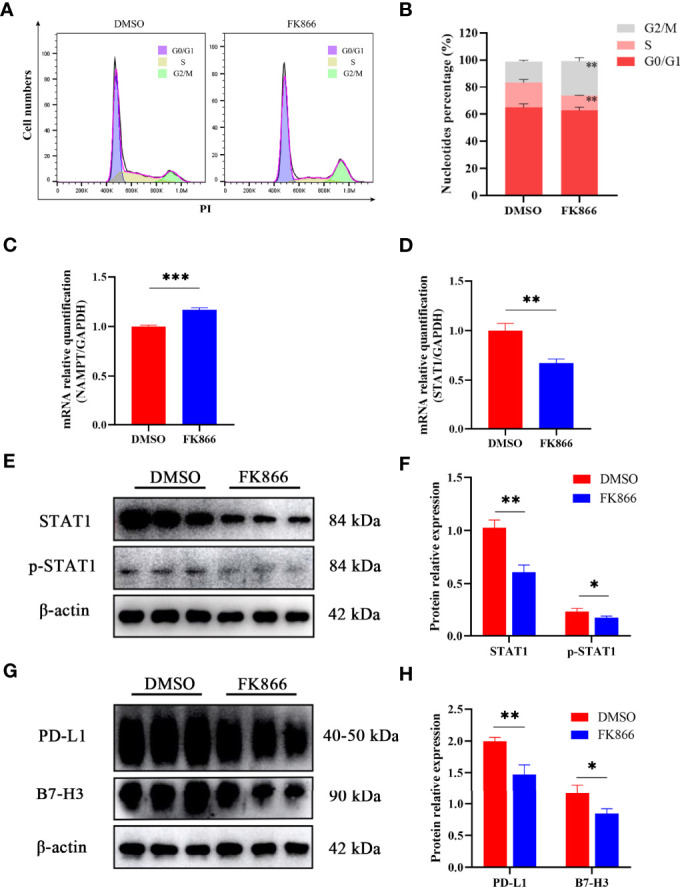
**(A, B)** Distribution of the cell cycle in IOMM cells treated with FK866 or DMSO. **(C, D) **Changes in NAMPT (NAMPT/GAPDH) and STAT1 (STAT1/GAPDH) mRNA relative expression in IOMM cells treated with FK866 or DMSO. **(E, F)** Changes in STAT1 (STAT1/β-actin) and p-STAT1 (p-STAT1/β-actin) protein relative expression in IOMM cells treated with FK866 or DMSO. **(G, H)** Changes in PD-L1 (PD-L1/β-actin) and B7-H3 (B7-H3/β-actin) protein relative expression in IOMM cells treated with FK866 or DMSO. *p < 0.05, **p < 0.01, ***p < 0.001.

To understand how FK866 influenced the proliferation of the IOMM cell line, flow cytometry was used to analyze the effect of FK866 on the cell cycle. The percentage of cells in the G2/M phase was significantly different between the FK866-treated group and the DMSO-treated group (p= 0.0025) (**Figures 5A, B**). The proportion of cells in the G2/M phase among FK866-treated IOMM cells had increased. In addition, a significant difference in the number of cells in the S phase was also observed between the FK866-treated group and the DMSO-treated group (p=0.0041) (**Figures 5A, B**). FK866 reduced the number of cells in the S phase.

### The NAMPT Inhibitor-FK866-Suppressed Immune Checkpoint Expression and Proliferation of IOMM Cells by Downregulating the Expression of STAT1

Reverse transcription-quantitative polymerase chain reaction (RT-qPCR) was preformed to detect the mRNA relative expression of NAMPT and STAT1. The results showed that FK866 can elevate the mRNA expression of NAMPT (**Figure 5C**) and reduce the mRNA expression of STAT1 (**Figure 5D**). And the mRNA relative expression of NAMPT and STAT1 showed significant differences between the DMSO group and the FK866 group (p=0.0003 and p=0.0022). Western blotting was preformed to detect the expression of STAT1 and p-STAT1. A significant difference was observed between the DMSO group and the FK866 group (p=0.0019 and p=0.0353) (**Figure 5F**). The results showed that FK866 reduced the expression of STAT1 and p-STAT1 (**Figure 5E**). Intriguingly, STAT1 can promote the expression of the immune checkpoint (PD-L1) (27, 34–36), which can lead to immune escape (26). Therefore, we performed Western blotting to detect the expression of immune checkpoint proteins. The expression of PD-L1 and B7-H3, indeed, showed significant differences between the DMSO group and the FK866 group (p=0.0053 and p=0.0250) (**Figure 5H**). FK866 may decrease the expression of PD-L1 and B7-H3 by downregulating STAT1 and p-STAT1 (**Figure 5G**).

### The Small-Molecular Inhibitor of NAMPT, FK866, Inhibited the Growth of Anaplastic Meningiomas and Immune Checkpoint Expression *In Vivo*

**Figure 6:**
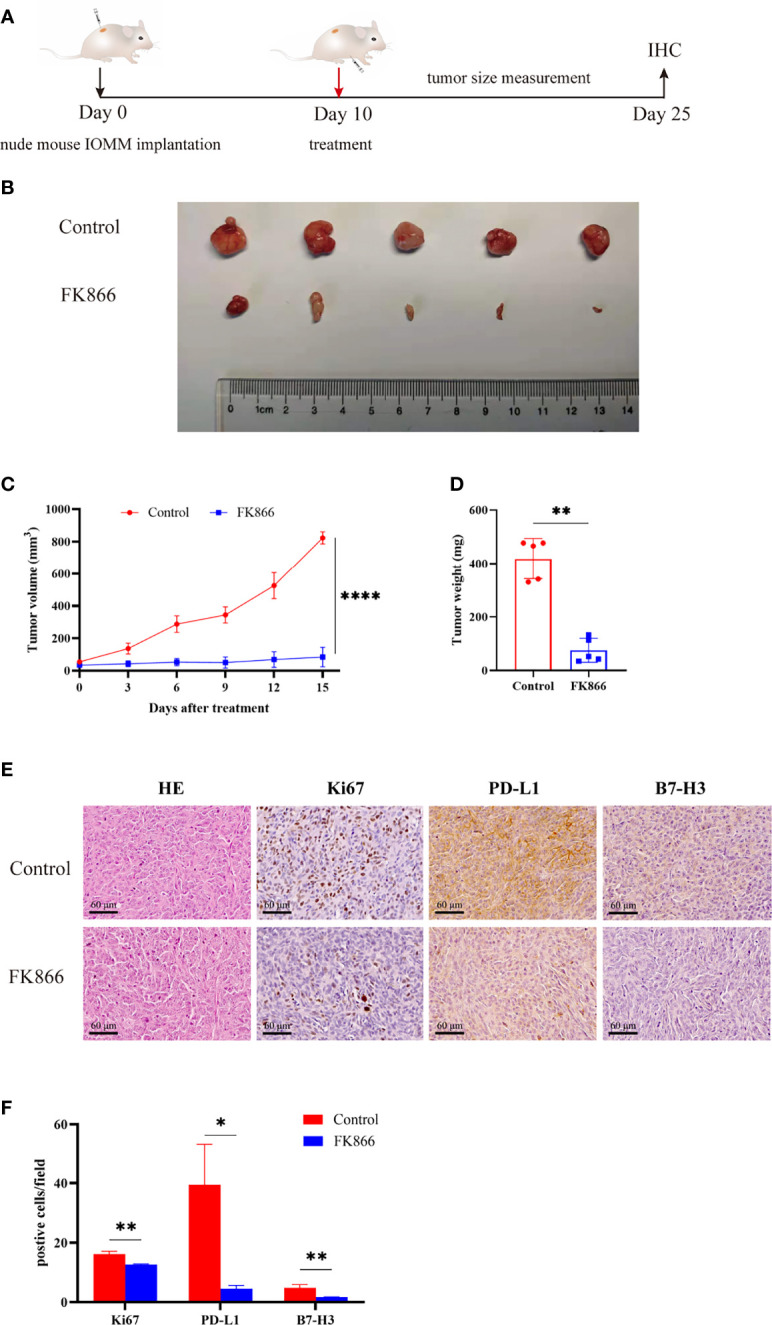
**(A, B)** Mice bearing IOMM meningiomas were treated with intraperitoneal injections of FK866 or DMSO on day 10, and the tumors were collected on day 25 (N=5/group).**(C)** The tumor volume at 3, 6, 9, 12 and 15 days after treatment. Tumor size (mm3) = [d2 x D]/2, d is the shortest diameter and D is the largest diameter. **(D)** The results of tumor weight analysis. **(E, F)** Immunohistochemical staining for Ki-67, PD-L1, B7-H3 in IOMM cell-derived meningiomas treated with DMSO and FK866. *p < 0.05, **p < 0.01, ****p < 0.0001.

To confirm the effect of FK866 on anaplastic meningiomas *in vivo*, we injected IOMM cells to generate an in vivo xenograft model (**Figure 6A**). Mice bearing IOMM subcutaneous xenograft tumors were randomized into two groups: (i) the Control and (ii) FK866 (5 mg/kg) groups (**Figure 6B**). Our results showed that tumor volumes were significantly lower in animals treated with FK866 than in animals treated with DMSO (p<0.0001) (**Figure 6C**). The tumor weight was significantly different between the FK866 group and the Control group (p=0.0079) (**Figure 6D**). Taken together, these results suggested that FK866 can be an effective curative therapy in anaplastic meningiomas. Moreover, changes of Ki-67, PD-L1 and B7-H3 expression *in vivo* were detected by immunohistochemical staining. The expression of Ki-67, PD-L1 and B7-H3 showed significant differences (p=0.0030, p=0.0113 and p=0.0077) (**Figure 6F**). The IOMM meningioma models treated with FK866 had low levels of Ki-67, PD-L1 and B7-H3 expression (**Figure 6E**). This suggested that FK866 can inhibit the growth of tumors and prohibit immune escape.

## Discussion

In this study, we used proteomic analysis to identify the differentially expressed proteins between fibrous meningiomas and anaplastic meningiomas. Then, NAMPT was identified as the critical protein during the tumorigenesis of anaplastic meningiomas. As the key rate-limiting enzyme in the salvage pathway of NAD synthesis, NAMPT has recently been shown to be a promoter of cancer (12–14). Sun M. Hong et al. reported that NAMPT can promote the proliferation of colorectal cancer cells (37). Zahra Hesari et al. reported that NAMPT promotes tumor progression and invasion of breast cancer cells (38). NAMPT can also facilitate pancreatic ductal adenocarcinoma cell growth (30). An increasing number of studies have proven that targeting NAMPT can be a feasible strategy for inhibiting tumor progression. However, targeting NAMPT to treat anaplastic meningiomas has not been reported. The NAMPT inhibitor, FK866, has shown anticancer activity in several tumor models (21–23) and has been tested in clinical trials (39). Li-Yuan Zhang et al. reported that FK866 can inhibit cell proliferation and induce G2/M cell-cycle arrest in glioblastoma cells (40), which corresponded to our results. We first validated that NAMPT plays a critical role in the tumorigenesis of anaplastic meningioma and can be a therapeutic target. Therefore, we posited that FK866 can suppress the progression of malignant meningiomas and experiments were performed to validate this assumption. In general, FK866 has a potential therapeutic effect on anaplastic meningiomas.

Previous studies have shown that the inhibition of NAMPT can dramatically reduce the activation of STAT1 (26). We wondered whether a NAMPT inhibitor suppresses the proliferation of IOMM cells by reducing STAT1. The results showed that inhibiting NAMPT by FK866 can reduce the expression of STAT1. The role of STAT1 in cancer is disputed. Most studies have suggested that STAT1 is a tumor suppresser (41). However, STAT1 can promote the progression of tumors in pleural mesothelioma (42), breast cancer (43) and head and neck cancer (44). Hongwei Lv et al. validated results showing that the NAMPT protein can facilitate the expression of STAT1. p-STAT1 expression was reduced in NAMPT-deficient cells or FK866-treated cells (26). In our study, the results showed that FK866 can reduce the expression of STAT1 and inhibit its activation. Intriguingly, a novel study revealed that STAT1 occupies a conserved element within the first intron of NAMPT. By binding this site, STAT1 can promote NAMPT expression in tumor-associated macrophages (45). It seems that there is a positive feedback mechanism involving STAT1 and NAMPT. Further study is needed to determine the relationship between these factors.

Recent studies have shown that NAMPT is a promoter of an immunosuppressive environment. Travelli et al. found that NAMPT can facilitate the mobilization of immature myeloid-derived suppressor cells (MDSCs), which can be suppressed by NAMPT inhibitors (19). Audrito V et al. found that NAMPT plays an important role into macrophage differentiation to the M2 phenotype and polarization into tumorigenesis-associated macrophages in leukemia (20). A number of previous studies have shown that NAMPT affects immune cells in tumors. However, the mechanism by which NAMPT regulates immune molecules in cancer cells needs to be determined. The relationship between NAMPT and immune checkpoints is still confusing. In glioblastoma, NAMPT inhibitors can upregulate PD-L1 in tumor cells, which makes combination therapy with NAMPT inhibitor and blockade of the PD-L1/PD-1 axis more efficient (46). However, Hongwei Lv et al. reported that inhibition of NAMPT reduced PD-L1 expression and that NAMPT deficiency impaired the therapeutic effect of PD-L1 checkpoint blockade on liver cancer in mice, which was further validated in melanoma samples obtained from patients (26). In our study, NAMPT inhibitors were used to suppress the growth of anaplastic meningiomas; at the same time, the expression of PD-L1 and B7-H3 was reduced, which means that FK866 can improve the tumor microenvironment and inhibit immune escape. Further work needs to be performed to determine the mechanism by which FK866 regulates the expression of immune checkpoints. This line of inquiry is important since the results may enable us to improve the efficacy of immune therapy for patients.

There are two major limitations in this study that could be addressed in future research. First, the study focused on the therapeutic effect of NAMPT inhibitor FK866 on anaplastic meningiomas and immune checkpoints expression. However, NAMPT knockdown experiments could be performed to investigate the mechanism about how NAMPT reduced STAT1 and immune checkpoints expression. Second, Although NAMPT was found at proteomic profiling and validated by western-blot and IHC, sample sizes could be increased for proteomic analysis to identify the more differentially expressed proteins at NAD metabolism.

In conclusion, we showed that anaplastic meningiomas have a high level of NAMPT expression, and we inhibited the growth of tumors *in vivo* and *in vitro* by applying an NAMPT inhibitor FK866. Notably, NAMPT inhibitors can reduce PD-L1 and B7-H3 expression in tumor cells, indicating their potential as targeted therapeutics for immune therapy.

## Data Availability Statement

The original contributions presented in the study are publicly available. This data can be found here: http://proteomecentral.proteomexchange.org; PXD032342.

## Ethics Statement

The studies involving human participants were reviewed and approved by the Ethics Committees of Beijing Tiantan Hospital (KY2021-158-01). The patients/participants provided their written informed consent to participate in this study. The animal study was reviewed and approved by Animals Welfare Ethics Committees of Beijing Neurosurgical Institute.

## Author Contributions

Study concept and design: YD and JF. Acquisition of data: YD, BH, YM, and JW. Analysis and interpretation of data: YD and YL. Drafting of the manuscript: YD and JF. Critical revision of the manuscript for important intellectual content: JF, NJ, ZW, and SH. Statistical analysis: YD and BH. Administrative, technical, and material support: YM, JW, SZ, DP, and HW. Study supervision: JF and SH. All authors contributed to the article and approved the submitted version.

## Funding

This work was supported by grants from the National Natural Science Foundation of China (81872052).

## Conflict of Interest

The authors declare that the research was conducted in the absence of any commercial or financial relationships that could be construed as a potential conflict of interest.

## Publisher’s Note

All claims expressed in this article are solely those of the authors and do not necessarily represent those of their affiliated organizations, or those of the publisher, the editors and the reviewers. Any product that may be evaluated in this article, or claim that may be made by its manufacturer, is not guaranteed or endorsed by the publisher.
