# Supplementary figures and images for: A Nicotinamide Phosphoribosyltransferase Inhibitor, FK866, Suppresses the Growth of Anaplastic Meningiomas and Inhibits Immune Checkpoint Expression by Regulating STAT1
Source: Front Oncol. 2022 Apr 20;12:836257. doi: 10.3389/fonc.2022.836257 (PMC9065474; doi:10.3389/fonc.2022.836257)

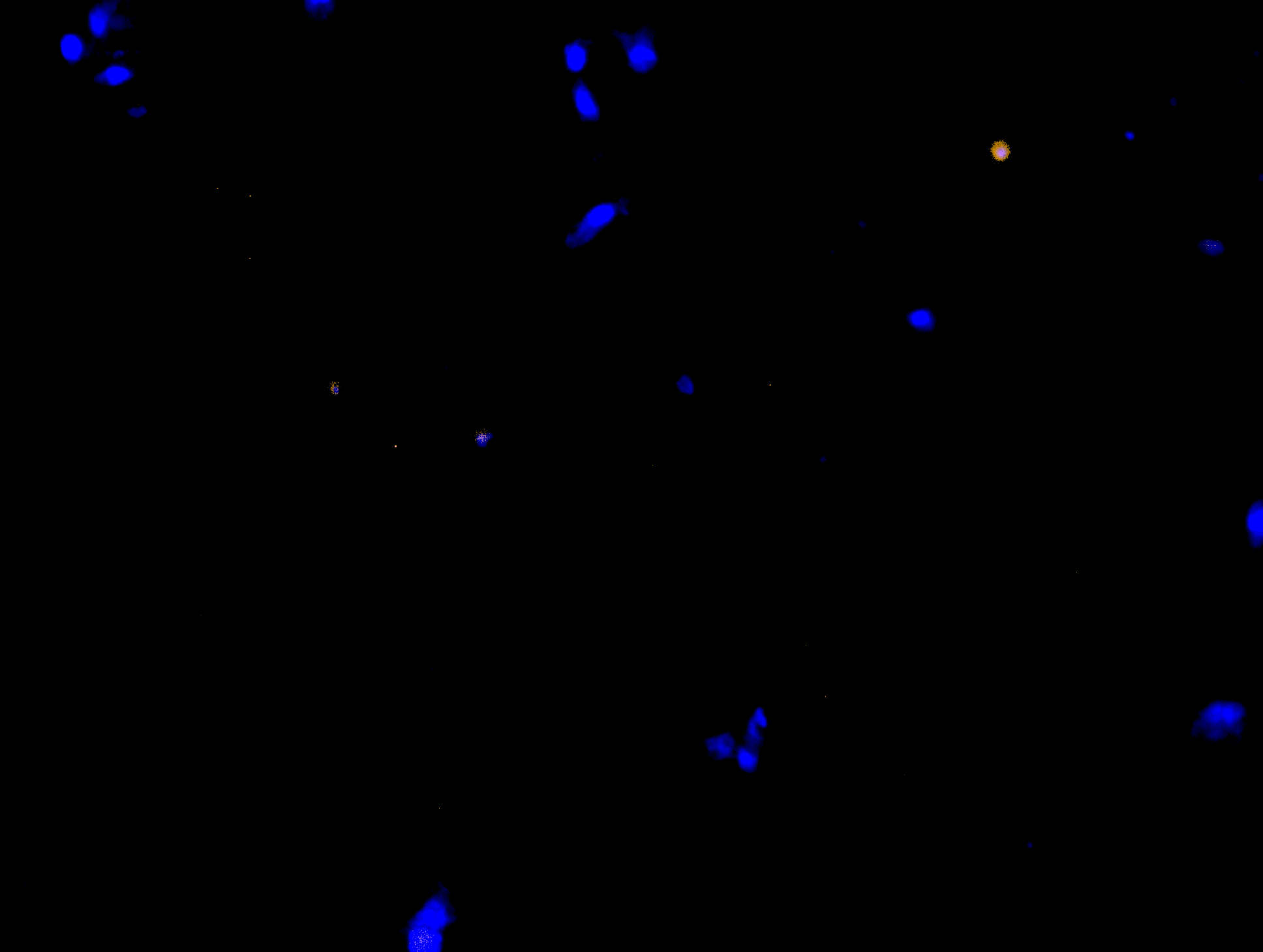

Supplement: Supplementary file 1 [file DataSheet_1.zip › edu Fig 4E/10xfk866 1.tif]

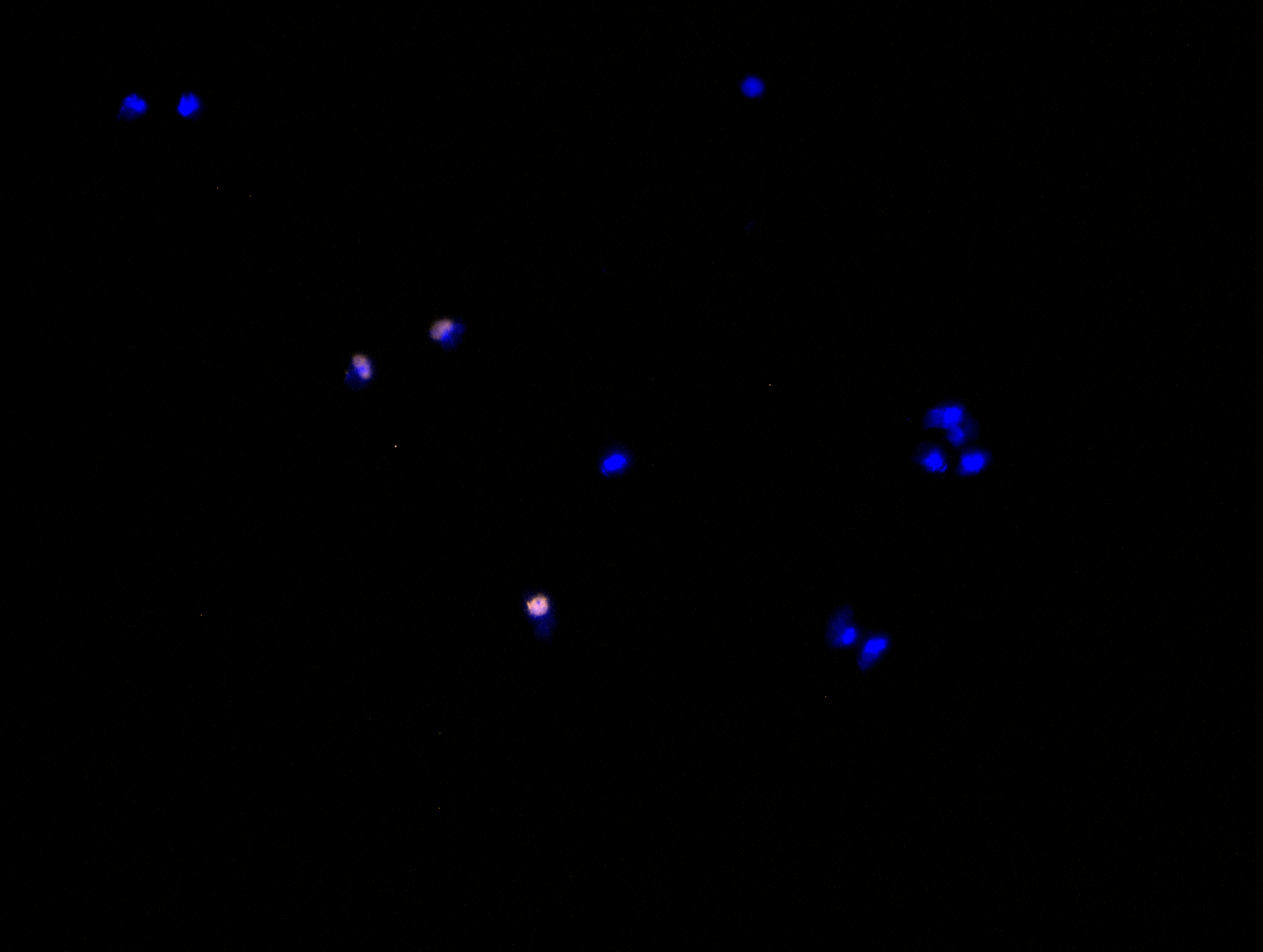

Supplement: Supplementary file 1 [file DataSheet_1.zip › edu Fig 4E/10xfk866 2.tif]

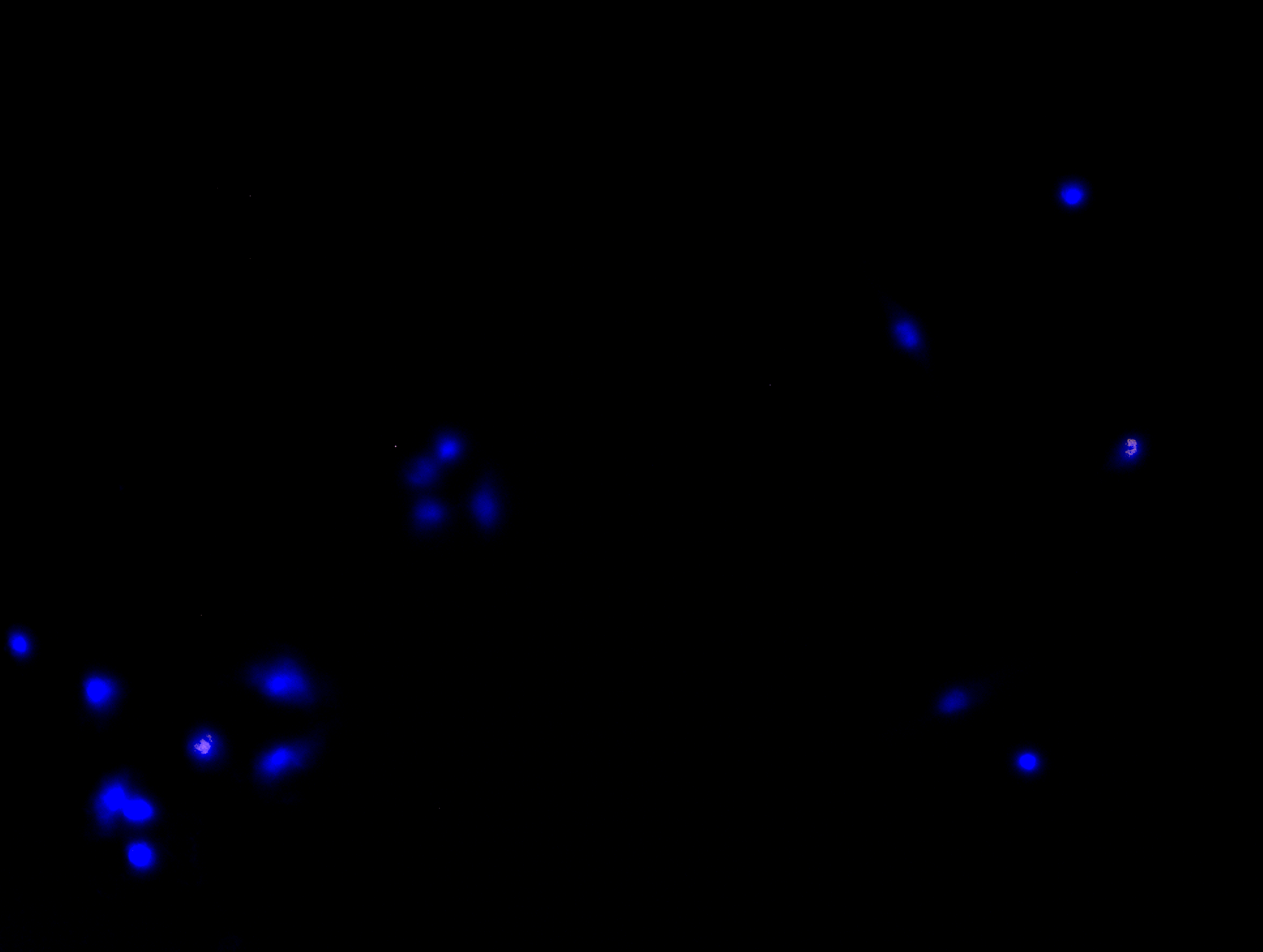

Supplement: Supplementary file 1 [file DataSheet_1.zip › edu Fig 4E/10xfk866 3.tif]

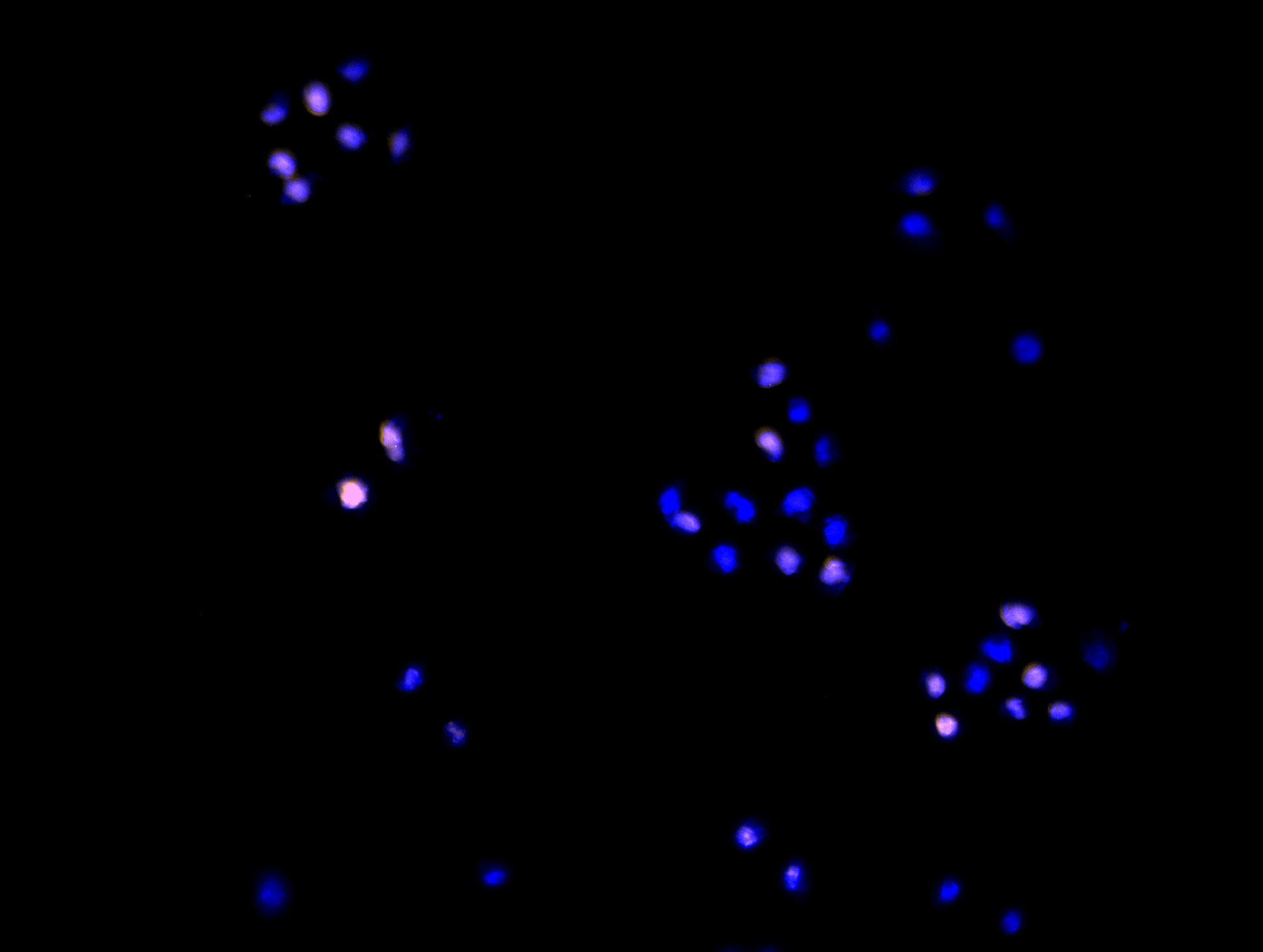

Supplement: Supplementary file 1 [file DataSheet_1.zip › edu Fig 4E/10xnc 1.tif]

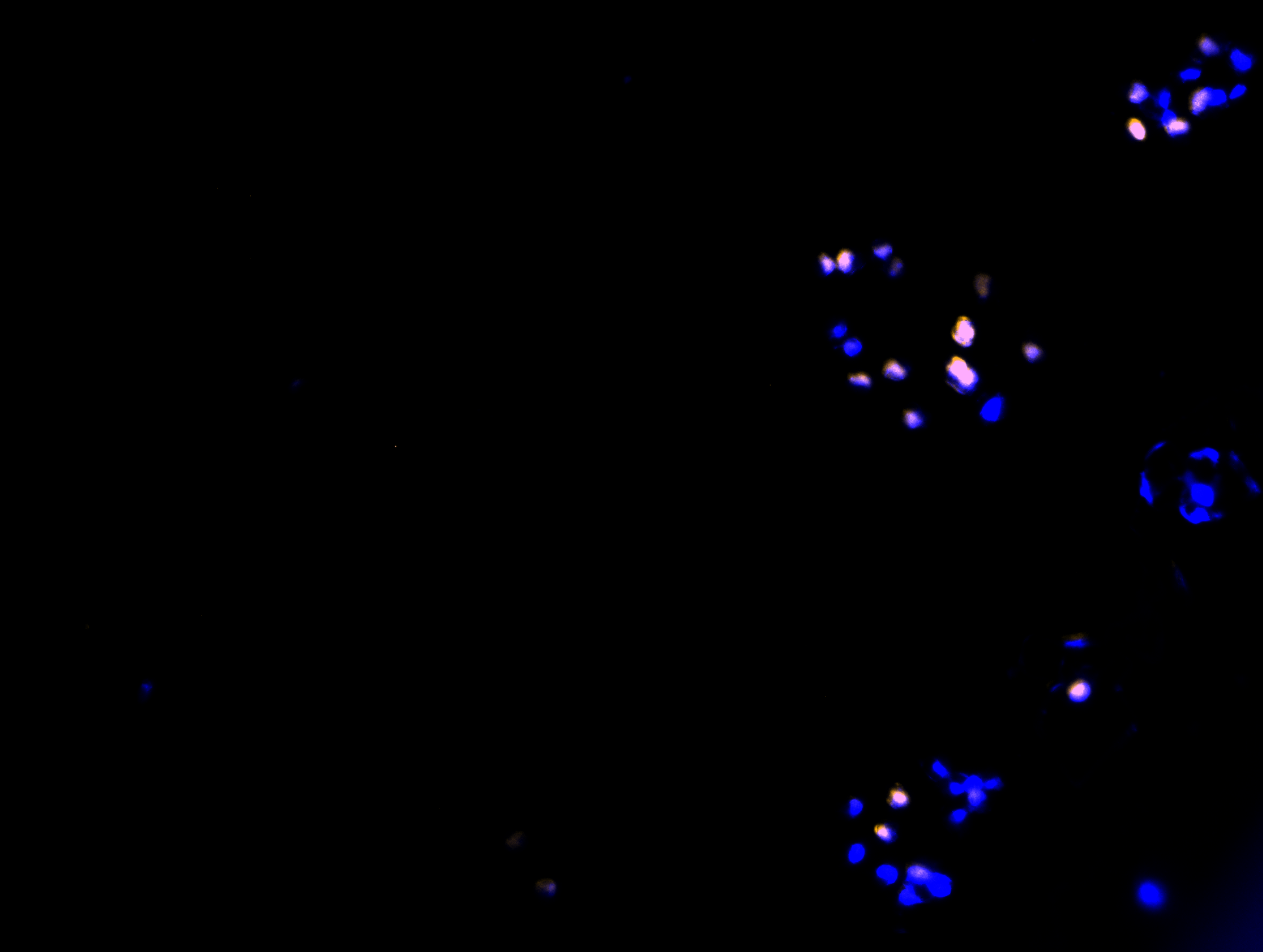

Supplement: Supplementary file 1 [file DataSheet_1.zip › edu Fig 4E/10xnc 2.tif]

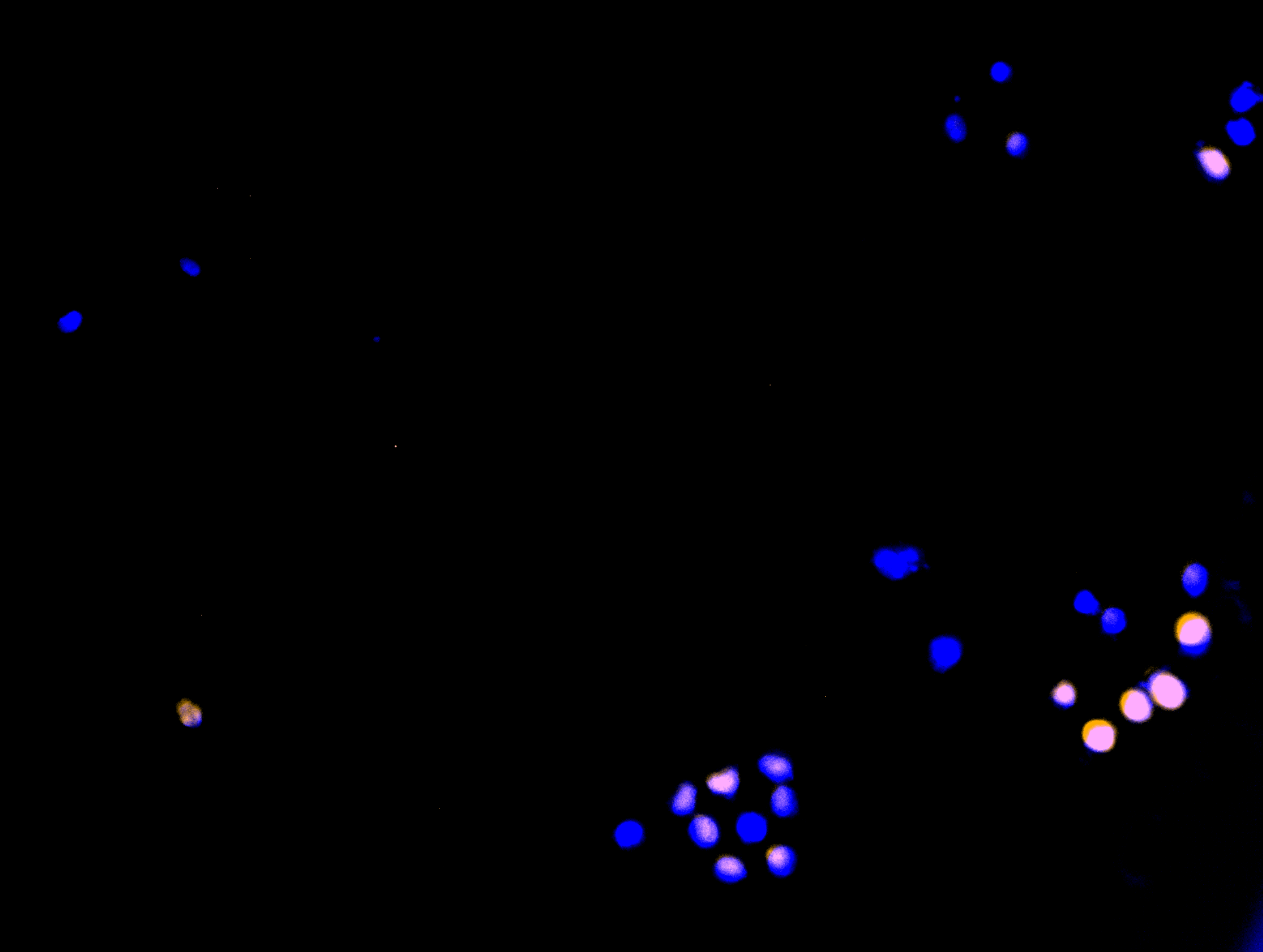

Supplement: Supplementary file 1 [file DataSheet_1.zip › edu Fig 4E/10xnc 3.tif]

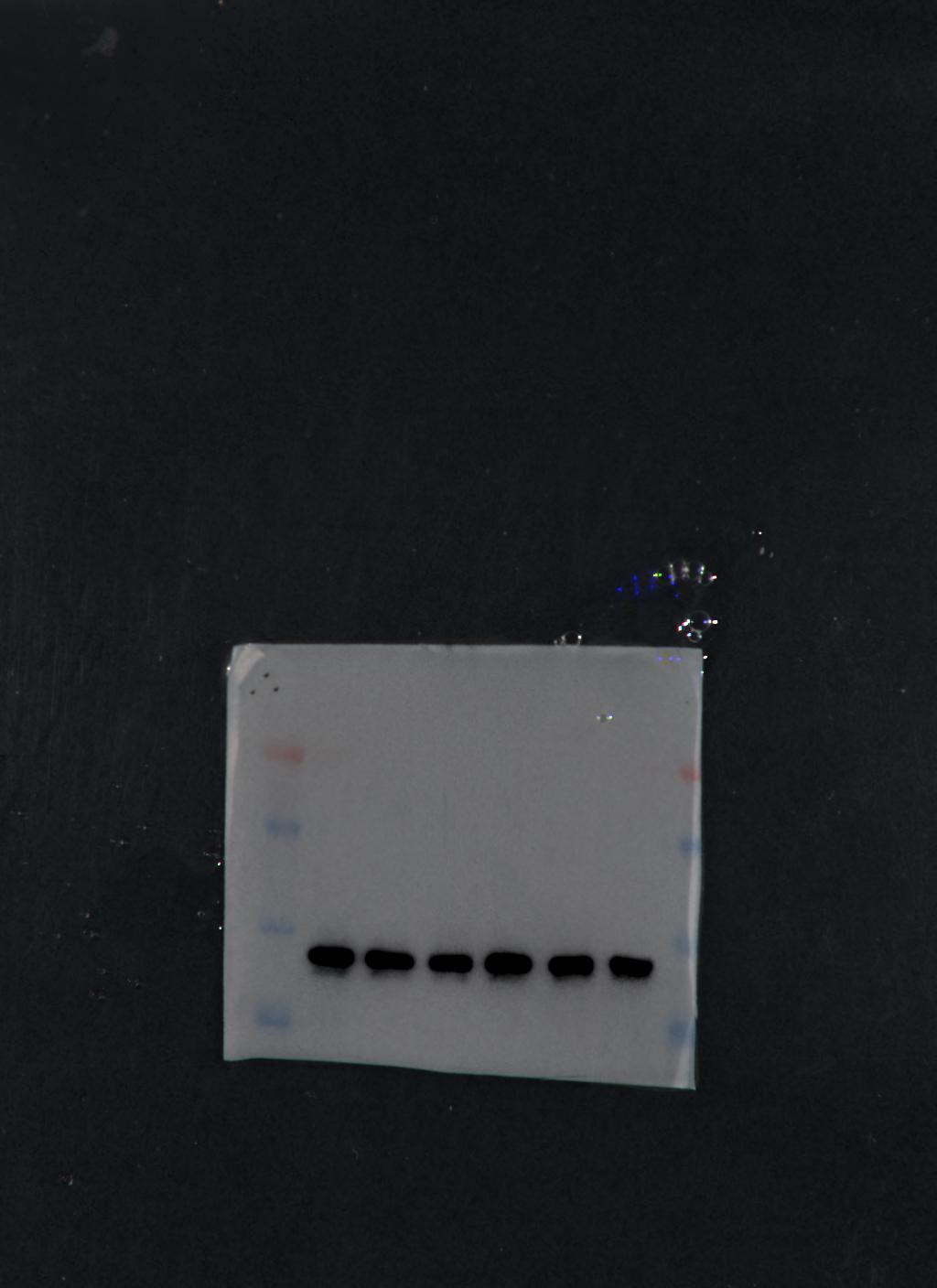

Supplement: Supplementary file 2 [file DataSheet_2.zip › wb Fig 5 C E/dyx-0512-Bactin-1 2021.05.12_20.15.44_Ch/dyx-0512-3-Bactin-1 2021.05.12_20.15.44_Ch+Marker.jpg]

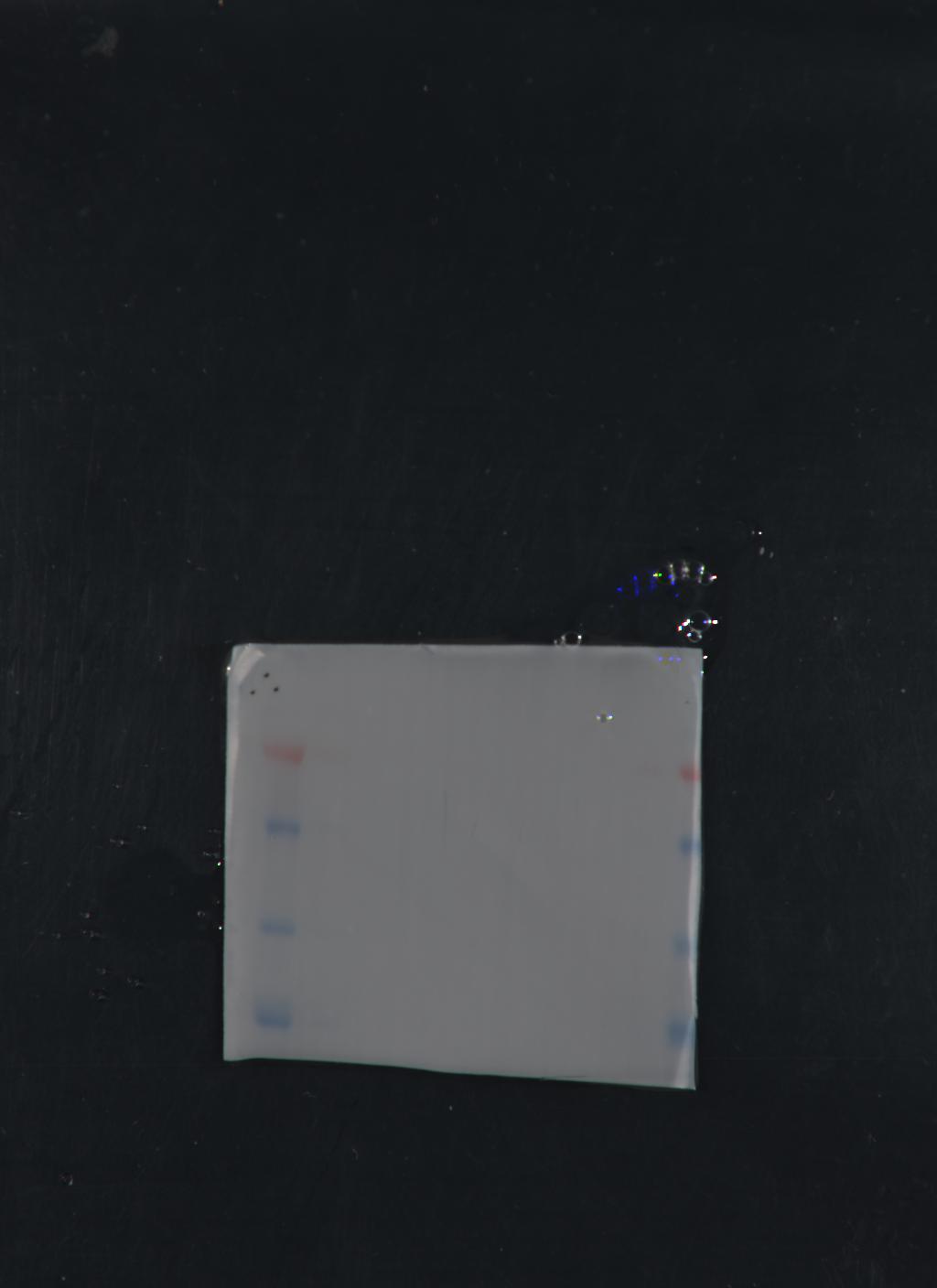

Supplement: Supplementary file 2 [file DataSheet_2.zip › wb Fig 5 C E/dyx-0512-Bactin-1 2021.05.12_20.15.44_Ch/dyx-0512-3-Bactin-1 2021.05.12_20.15.44_Ch-Marker.jpg]

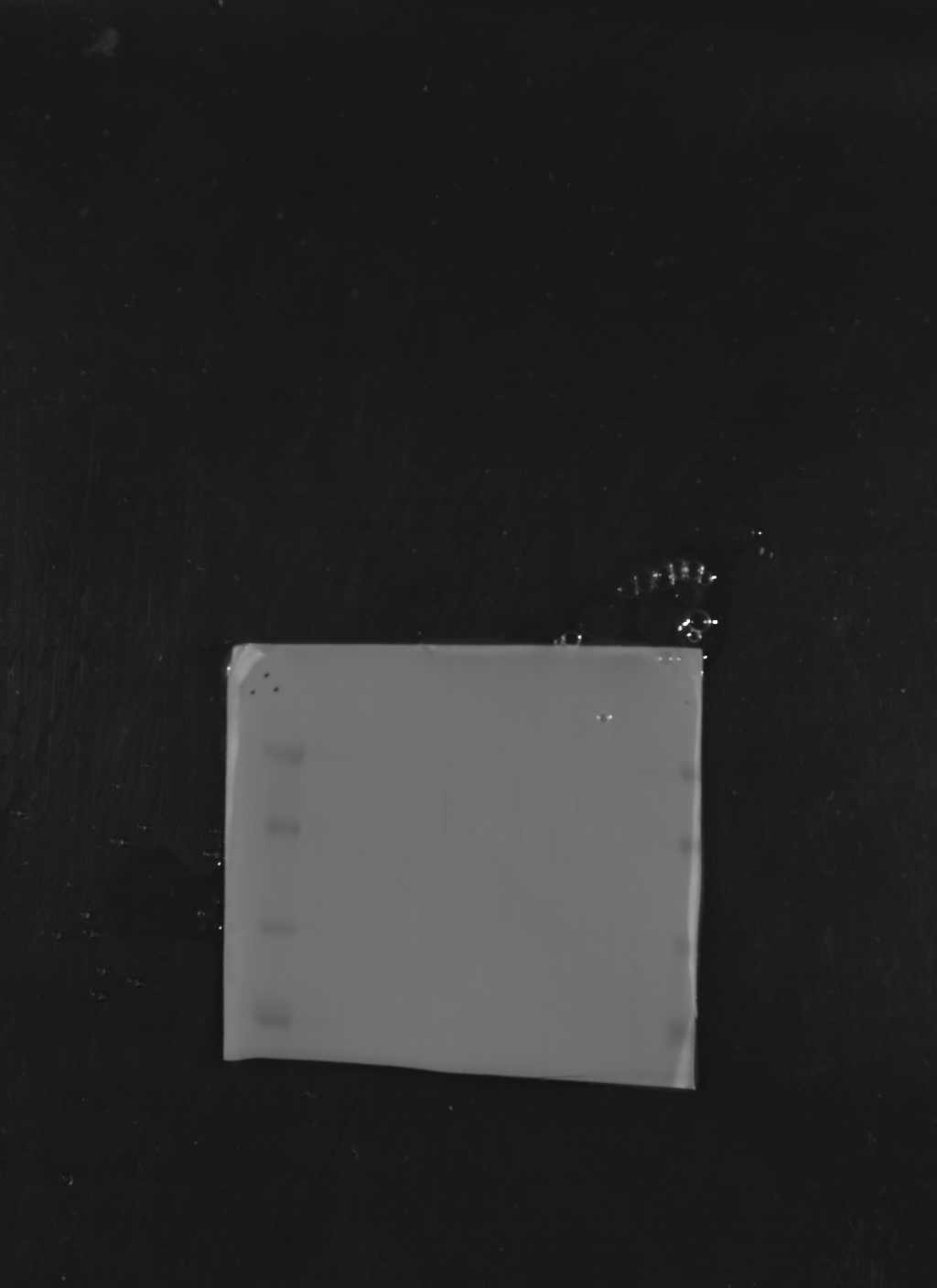

Supplement: Supplementary file 2 [file DataSheet_2.zip › wb Fig 5 C E/dyx-0512-Bactin-1 2021.05.12_20.15.44_Ch/dyx-0512-3-Bactin-1 2021.05.12_20.15.44_Ch-Marker.tif]

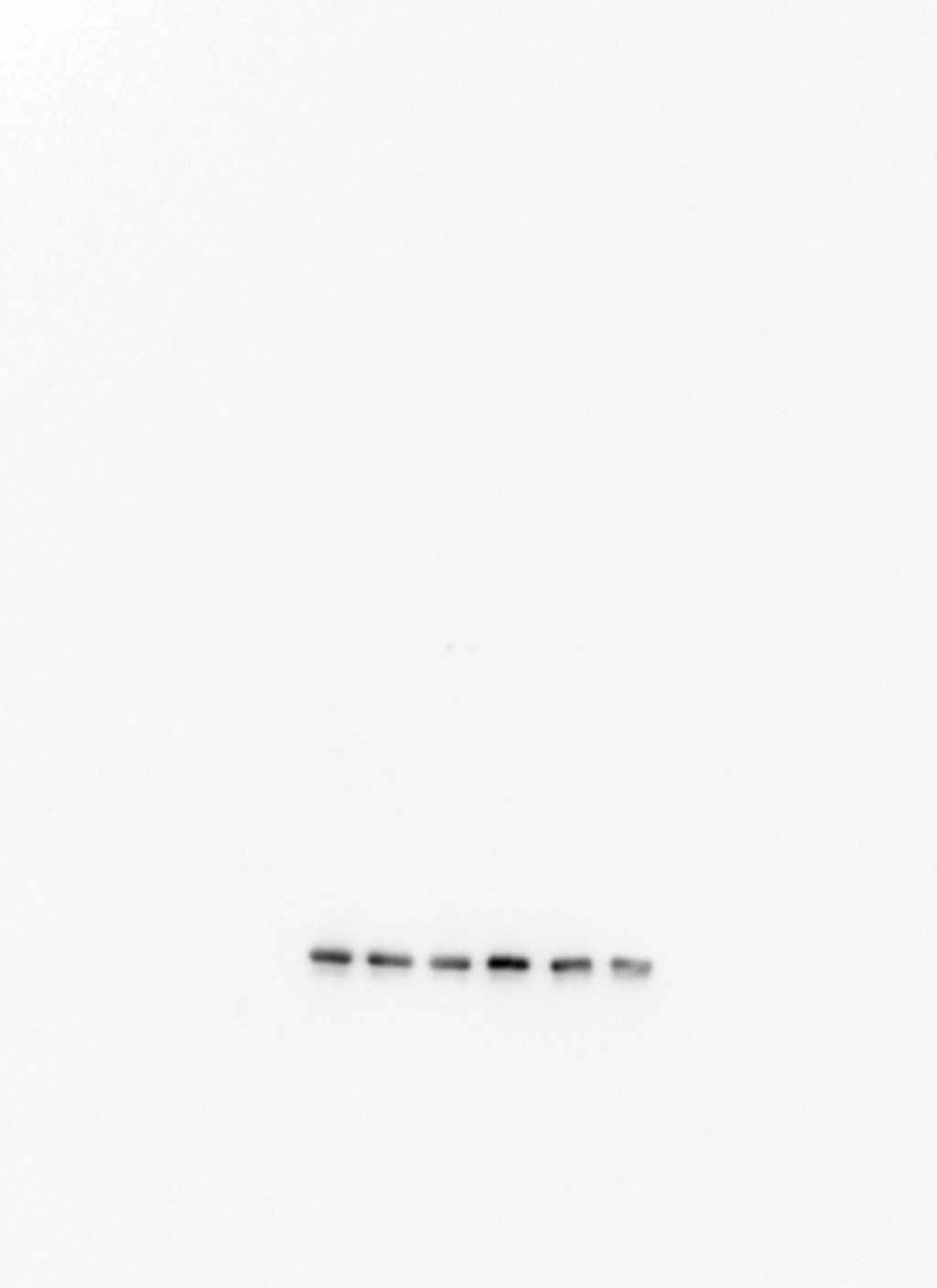

Supplement: Supplementary file 2 [file DataSheet_2.zip › wb Fig 5 C E/dyx-0512-Bactin-1 2021.05.12_20.15.44_Ch/dyx-0512-3-Bactin-1 2021.05.12_20.15.44_Ch.tif]

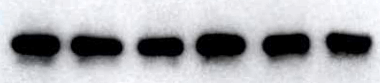

Supplement: Supplementary file 2 [file DataSheet_2.zip › wb Fig 5 C E/dyx-0512-Bactin-1 2021.05.12_20.15.44_Ch/F ACTIN.tif]

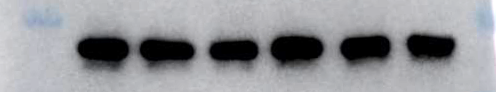

Supplement: Supplementary file 2 [file DataSheet_2.zip › wb Fig 5 C E/dyx-0512-Bactin-1 2021.05.12_20.15.44_Ch/psdyx-0512-3-Bactin-1 2021.05.12_20.15.44_Ch+Marker.tif]

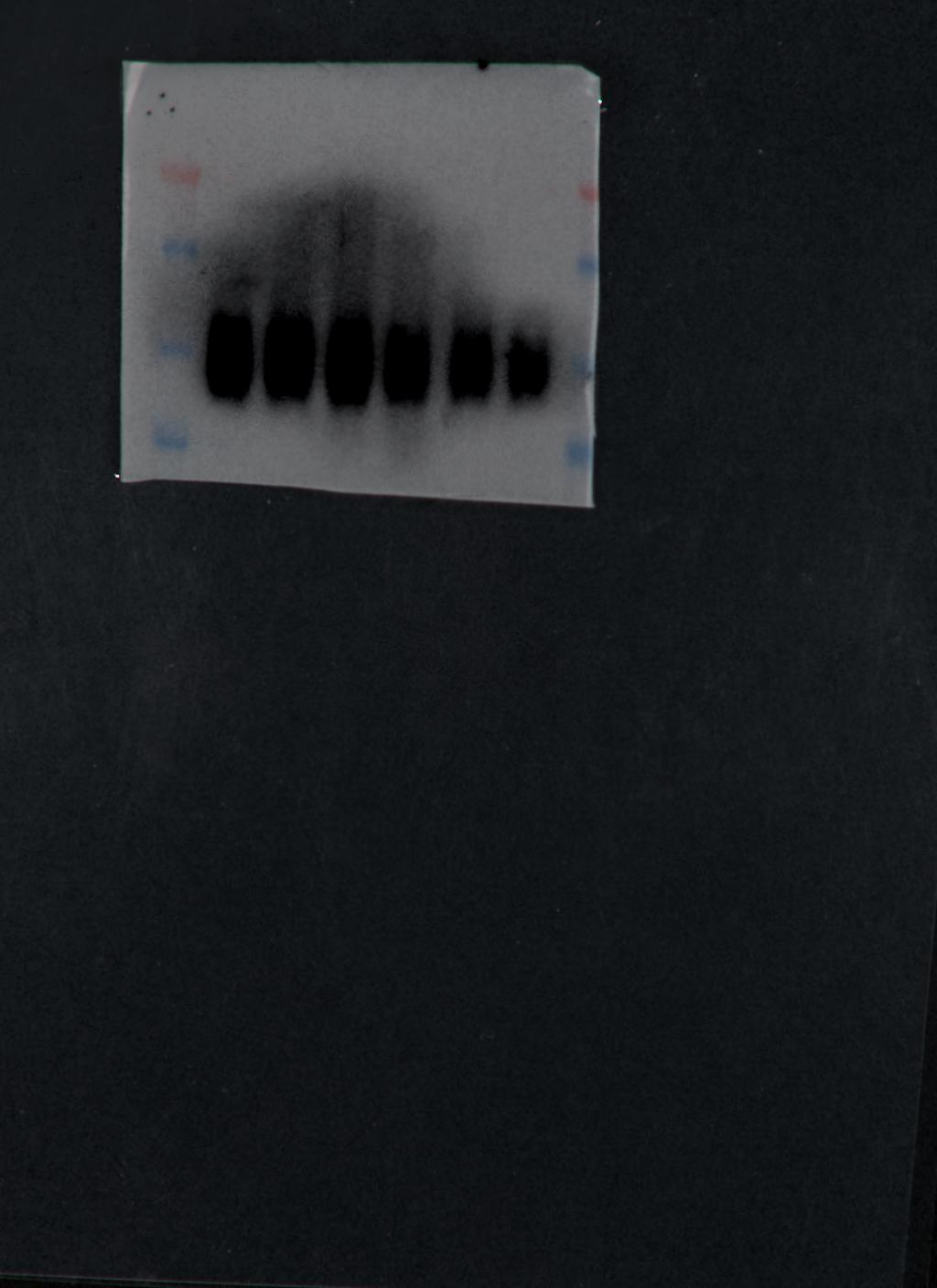

Supplement: Supplementary file 2 [file DataSheet_2.zip › wb Fig 5 C E/dyx-0512-PDL1-2 2021.05.12_14.32.09_Ch/dyx-0512-3-PDL1-2 2021.05.12_14.32.09_Ch+Marker.jpg]

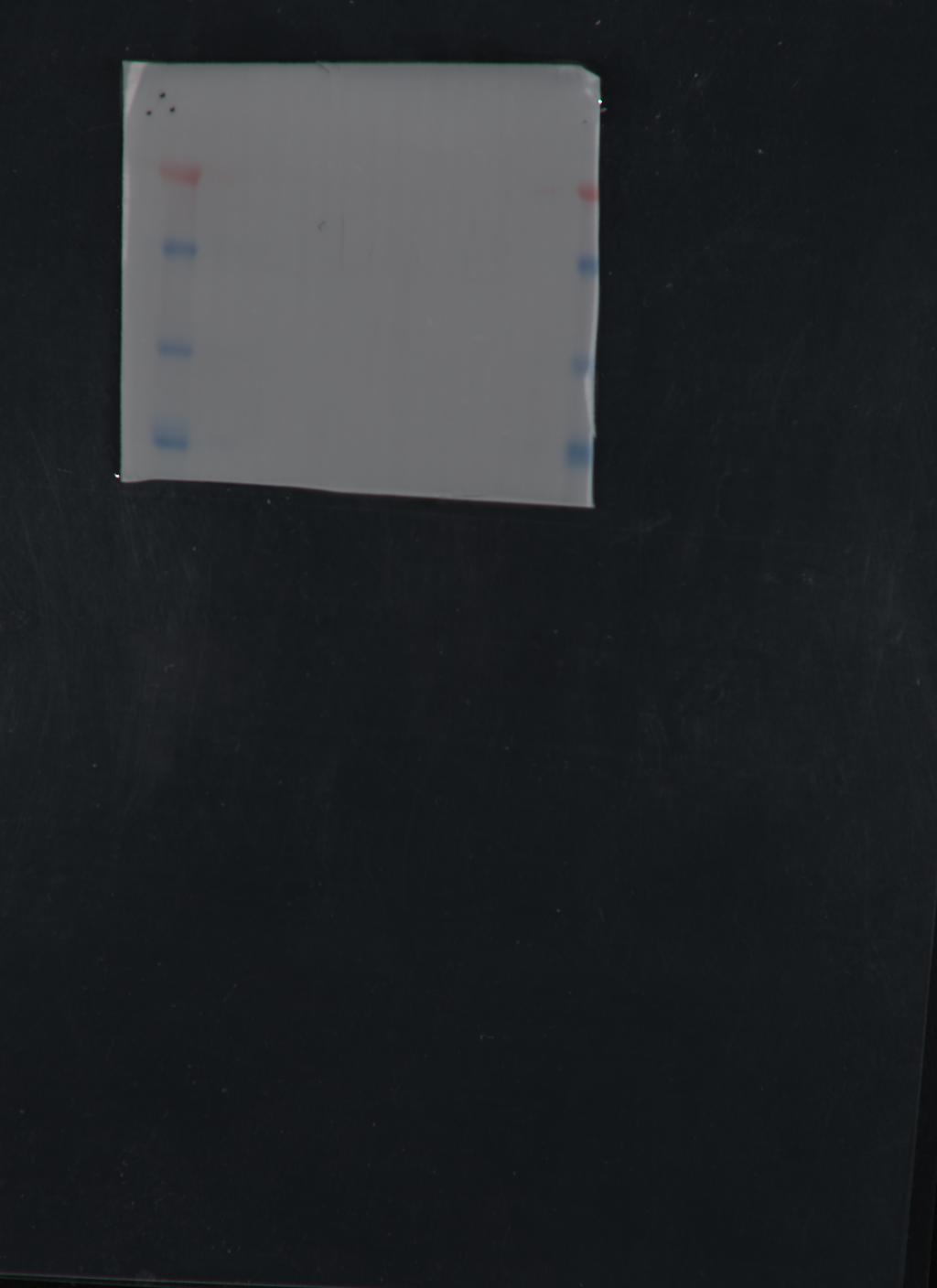

Supplement: Supplementary file 2 [file DataSheet_2.zip › wb Fig 5 C E/dyx-0512-PDL1-2 2021.05.12_14.32.09_Ch/dyx-0512-3-PDL1-2 2021.05.12_14.32.09_Ch-Marker.jpg]

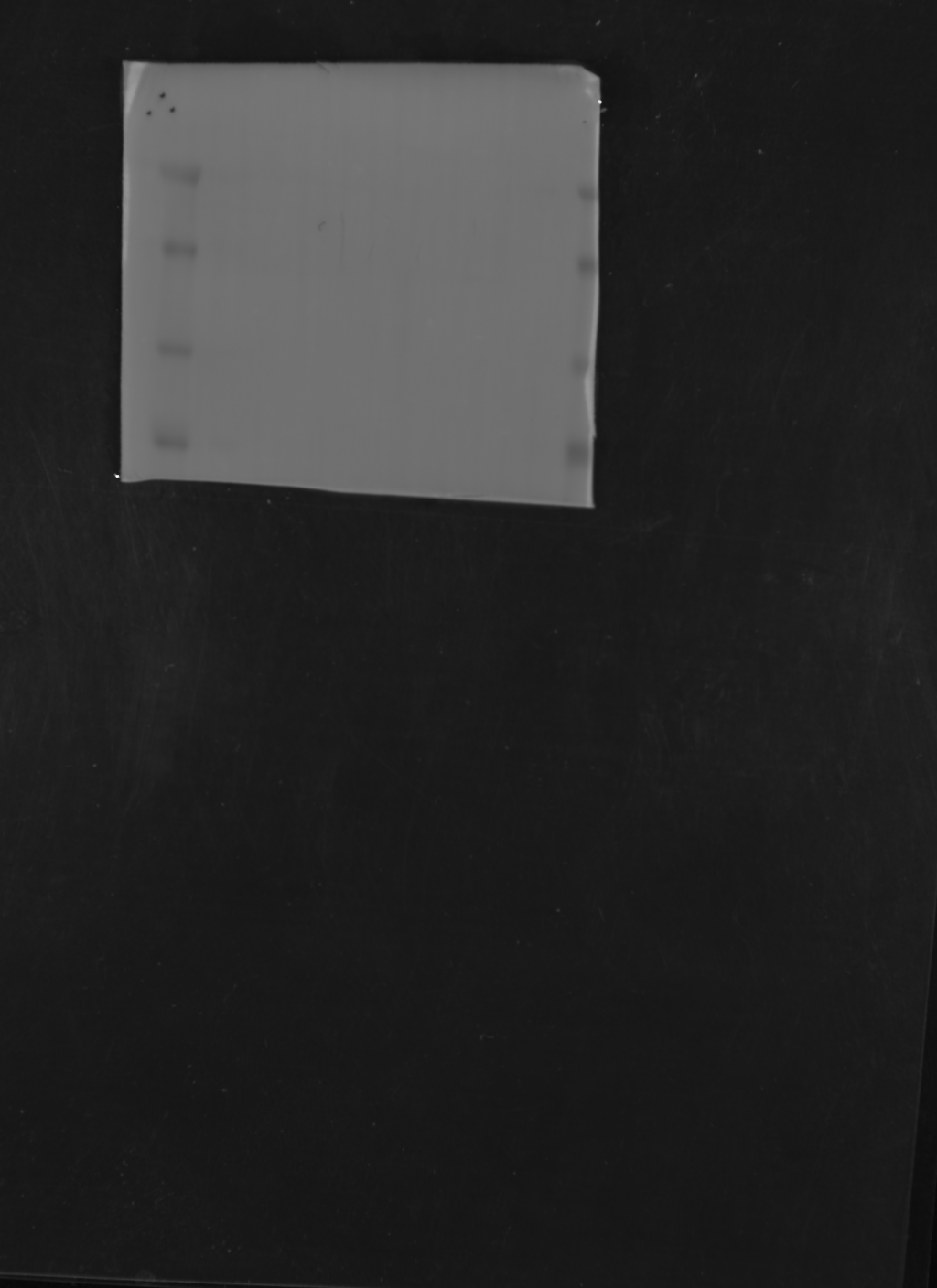

Supplement: Supplementary file 2 [file DataSheet_2.zip › wb Fig 5 C E/dyx-0512-PDL1-2 2021.05.12_14.32.09_Ch/dyx-0512-3-PDL1-2 2021.05.12_14.32.09_Ch-Marker.tif]

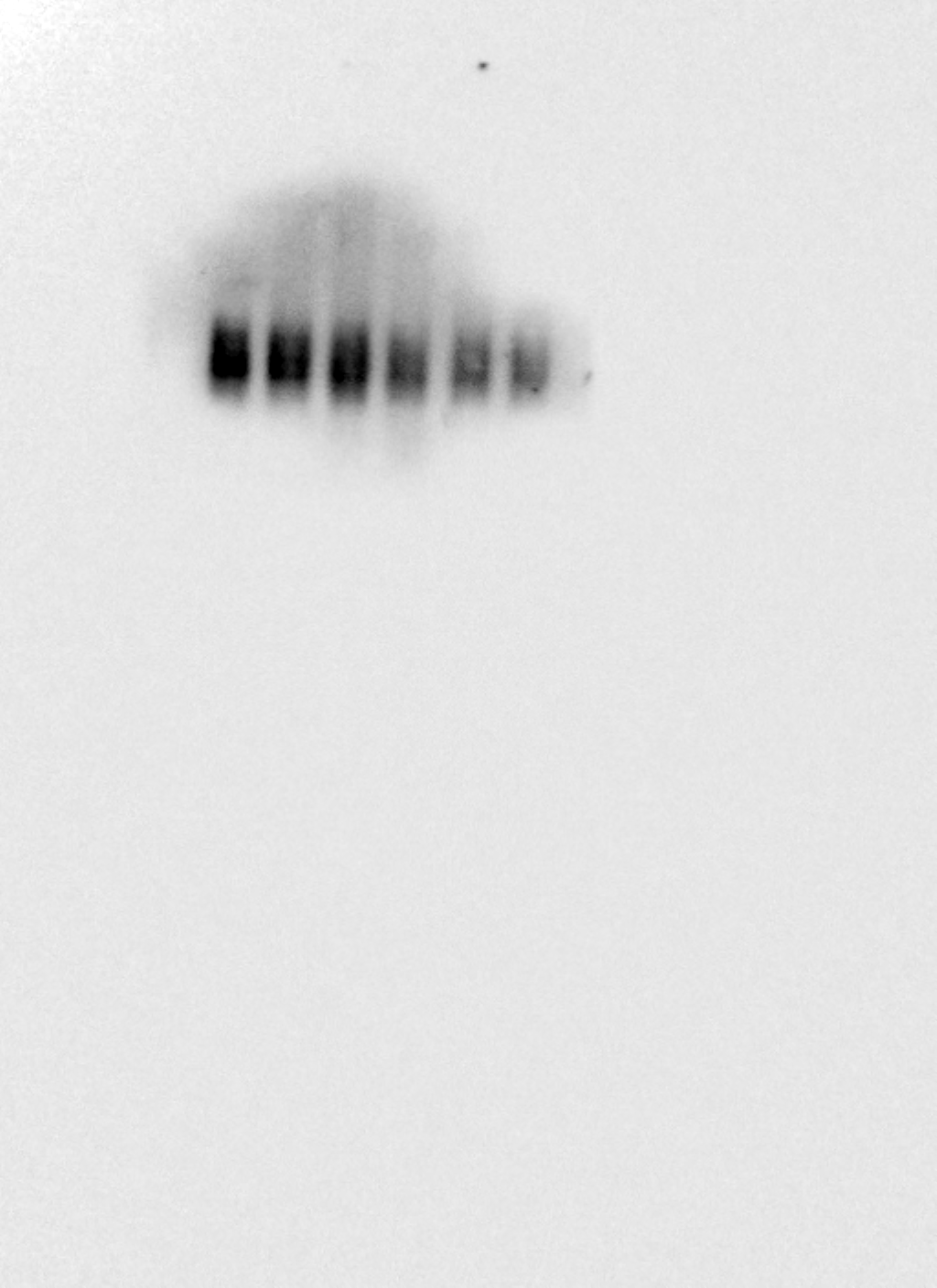

Supplement: Supplementary file 2 [file DataSheet_2.zip › wb Fig 5 C E/dyx-0512-PDL1-2 2021.05.12_14.32.09_Ch/dyx-0512-3-PDL1-2 2021.05.12_14.32.09_Ch.tif]

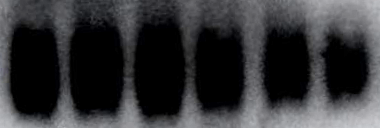

Supplement: Supplementary file 2 [file DataSheet_2.zip › wb Fig 5 C E/dyx-0512-PDL1-2 2021.05.12_14.32.09_Ch/F PDL1.tif]

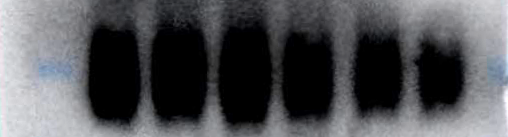

Supplement: Supplementary file 2 [file DataSheet_2.zip › wb Fig 5 C E/dyx-0512-PDL1-2 2021.05.12_14.32.09_Ch/psdyx-0512-3-PDL1-2 2021.05.12_14.32.09_Ch+Marker.tif]

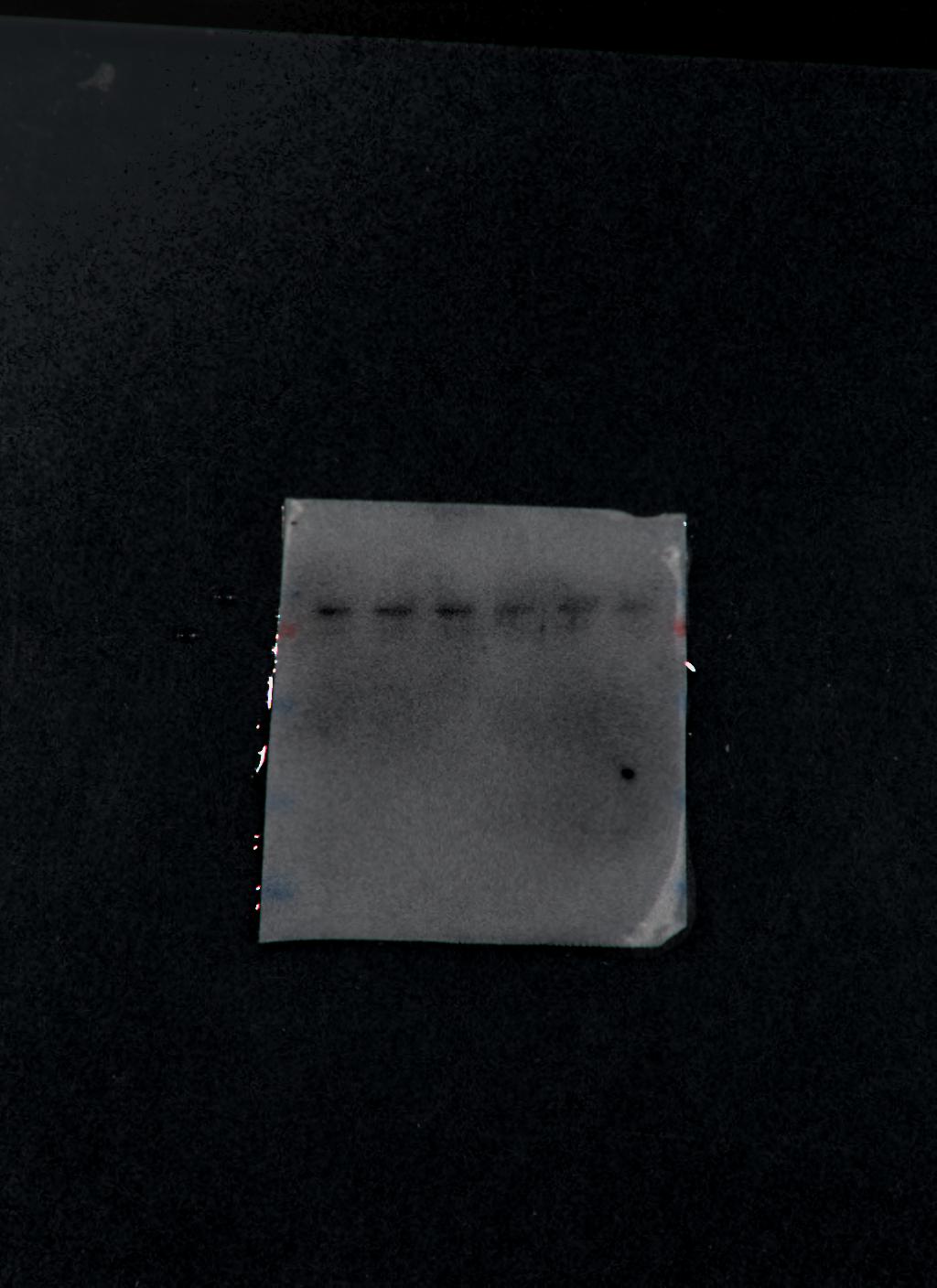

Supplement: Supplementary file 2 [file DataSheet_2.zip › wb Fig 5 C E/dyx-0512-PSTAT1-1 2021.05.12_14.49.13_Ch/dyx-0512-3-PSTAT1-1 2021.05.12_14.49.13_Ch+Marker.jpg]

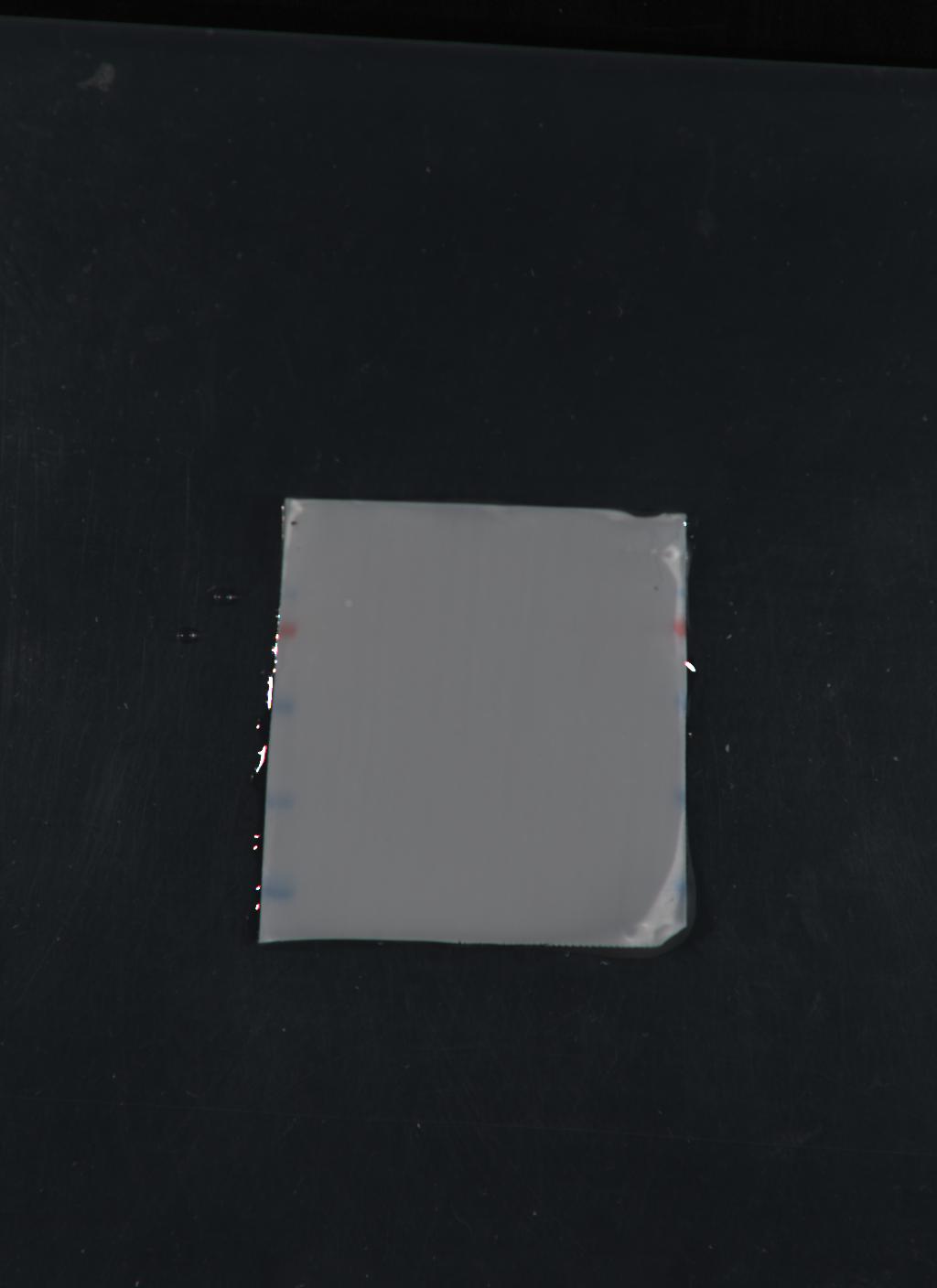

Supplement: Supplementary file 2 [file DataSheet_2.zip › wb Fig 5 C E/dyx-0512-PSTAT1-1 2021.05.12_14.49.13_Ch/dyx-0512-3-PSTAT1-1 2021.05.12_14.49.13_Ch-Marker.jpg]

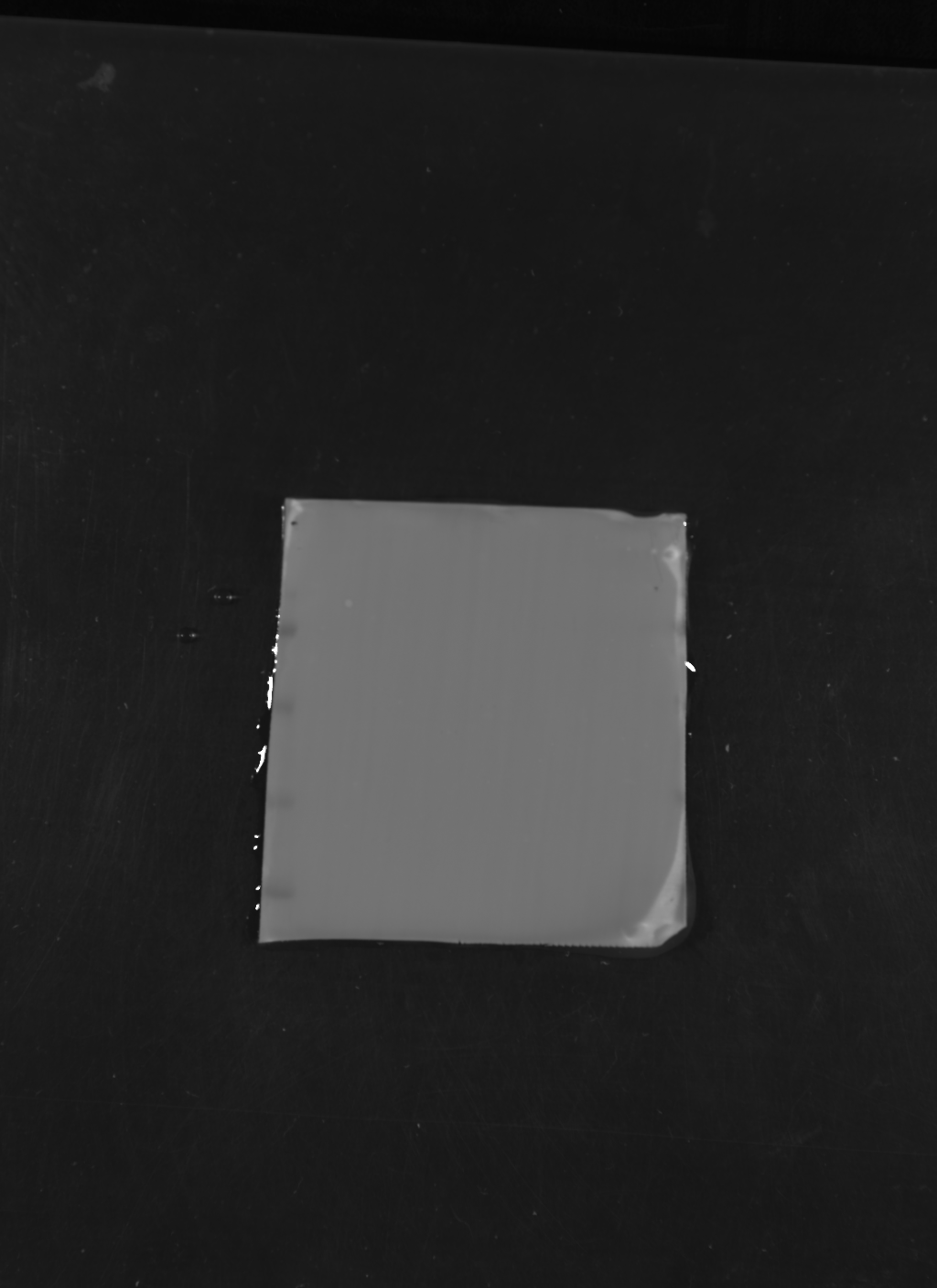

Supplement: Supplementary file 2 [file DataSheet_2.zip › wb Fig 5 C E/dyx-0512-PSTAT1-1 2021.05.12_14.49.13_Ch/dyx-0512-3-PSTAT1-1 2021.05.12_14.49.13_Ch-Marker.tif]

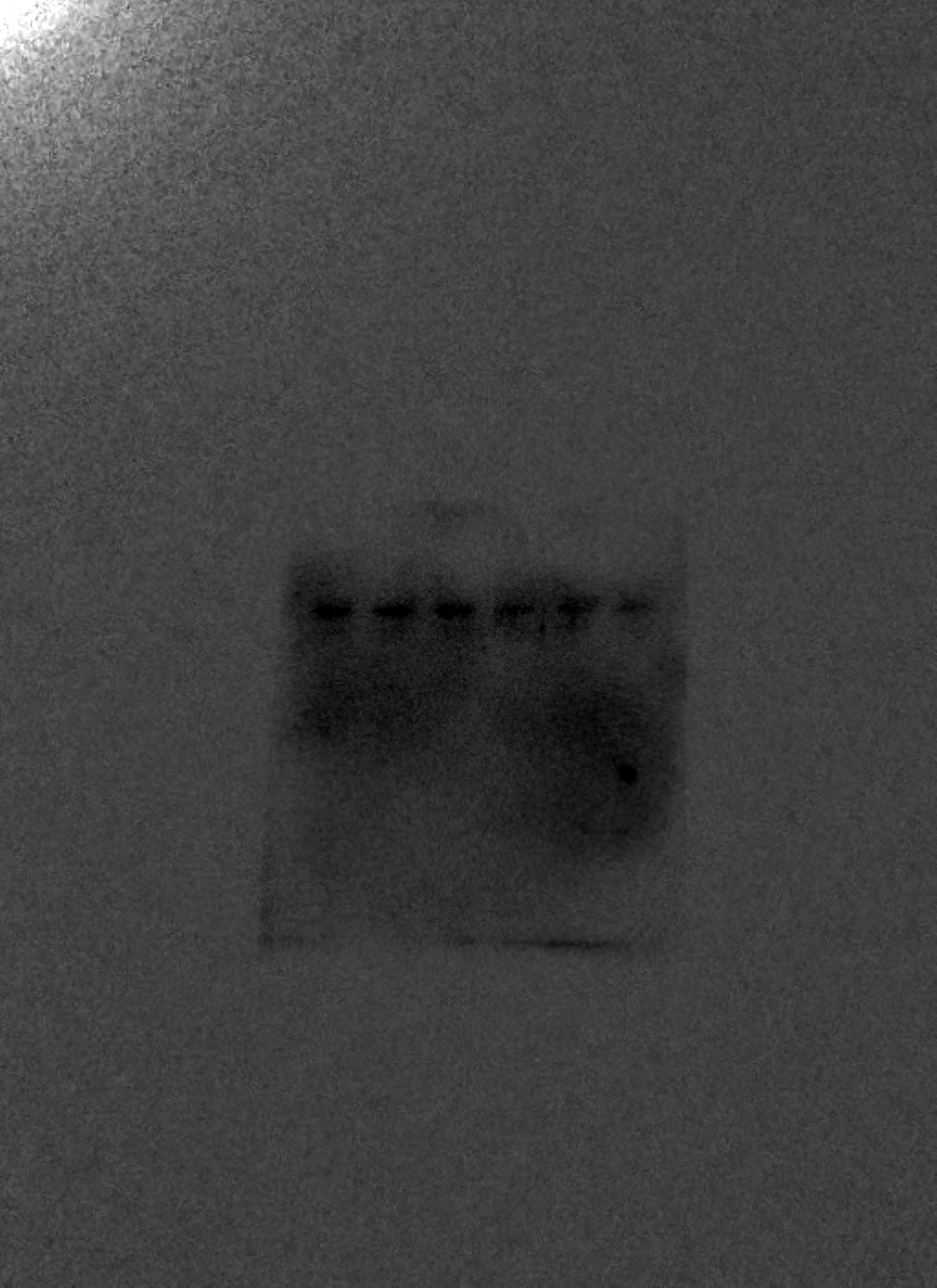

Supplement: Supplementary file 2 [file DataSheet_2.zip › wb Fig 5 C E/dyx-0512-PSTAT1-1 2021.05.12_14.49.13_Ch/dyx-0512-3-PSTAT1-1 2021.05.12_14.49.13_Ch.tif]

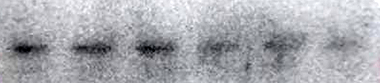

Supplement: Supplementary file 2 [file DataSheet_2.zip › wb Fig 5 C E/dyx-0512-PSTAT1-1 2021.05.12_14.49.13_Ch/F pstat1.tif]

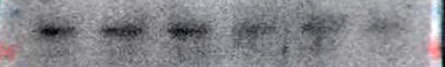

Supplement: Supplementary file 2 [file DataSheet_2.zip › wb Fig 5 C E/dyx-0512-PSTAT1-1 2021.05.12_14.49.13_Ch/PSdyx-0512-3-PSTAT1-1 2021.05.12_14.49.13_Ch+Marker.tif]

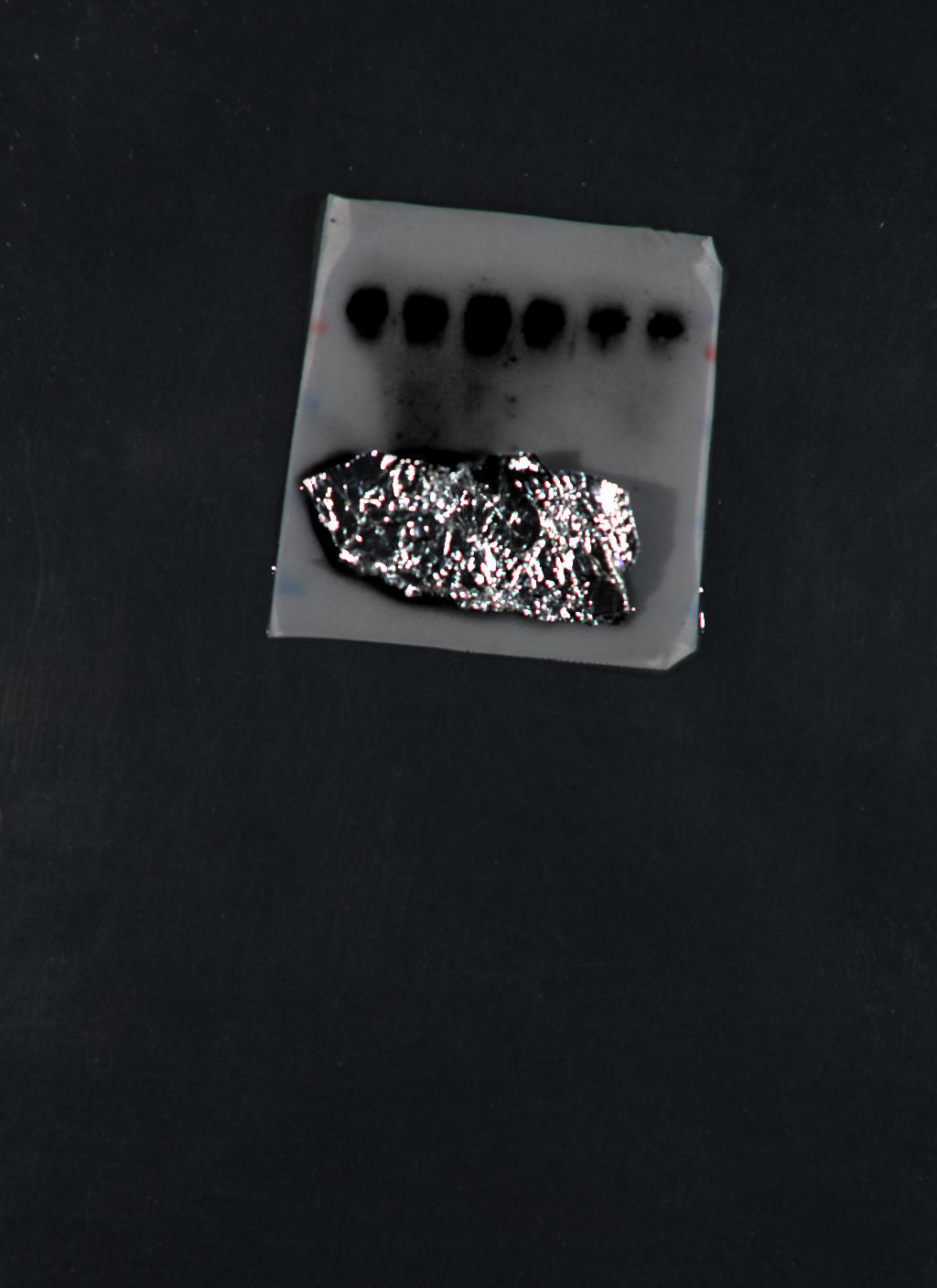

Supplement: Supplementary file 2 [file DataSheet_2.zip › wb Fig 5 C E/dyx-0514-B7H3-1 2021.05.13_16.16.53_Ch/dyx-0514-1-B7H3-1 2021.05.13_16.16.53_Ch+Marker.jpg]

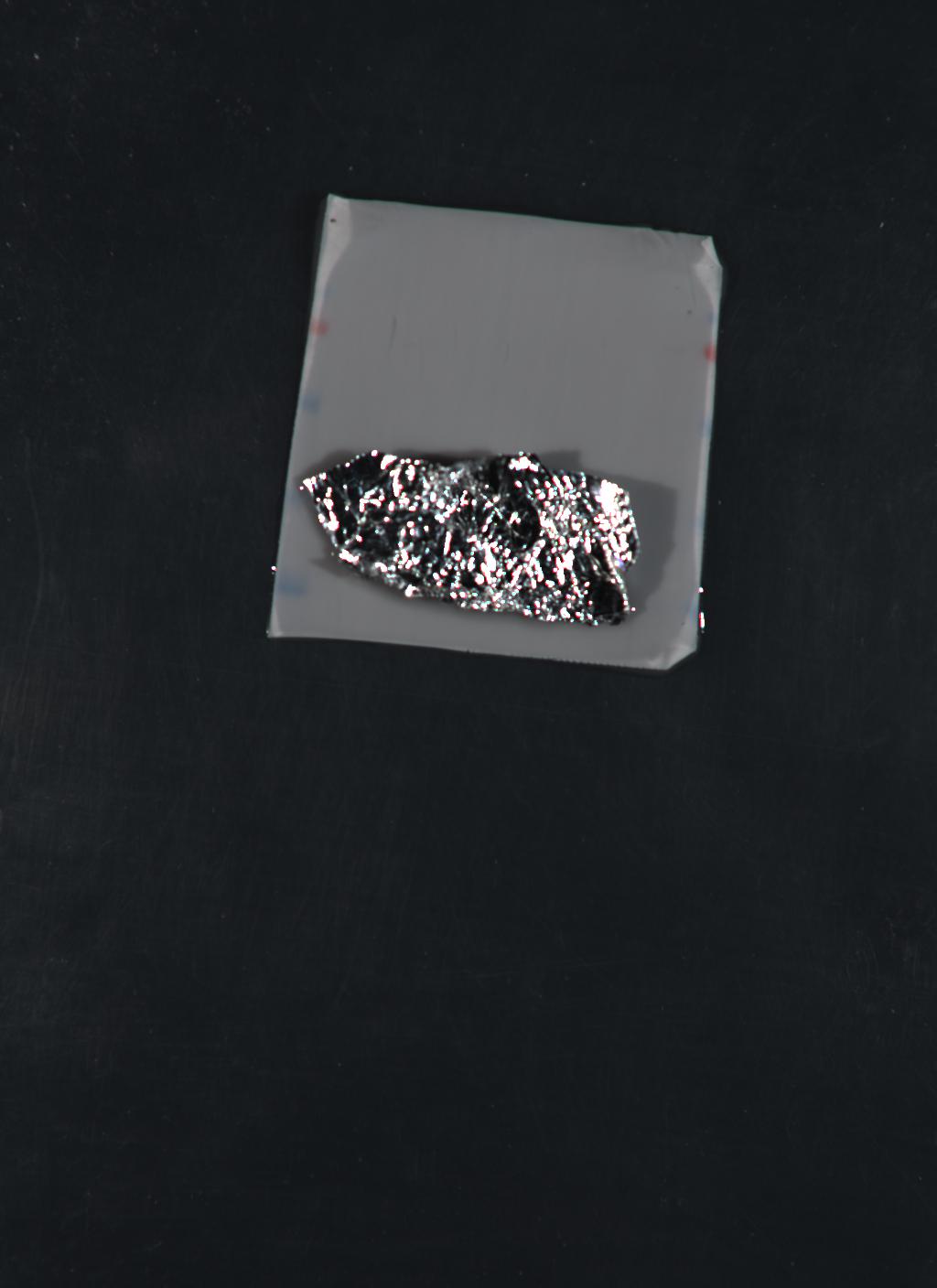

Supplement: Supplementary file 2 [file DataSheet_2.zip › wb Fig 5 C E/dyx-0514-B7H3-1 2021.05.13_16.16.53_Ch/dyx-0514-1-B7H3-1 2021.05.13_16.16.53_Ch-Marker.jpg]

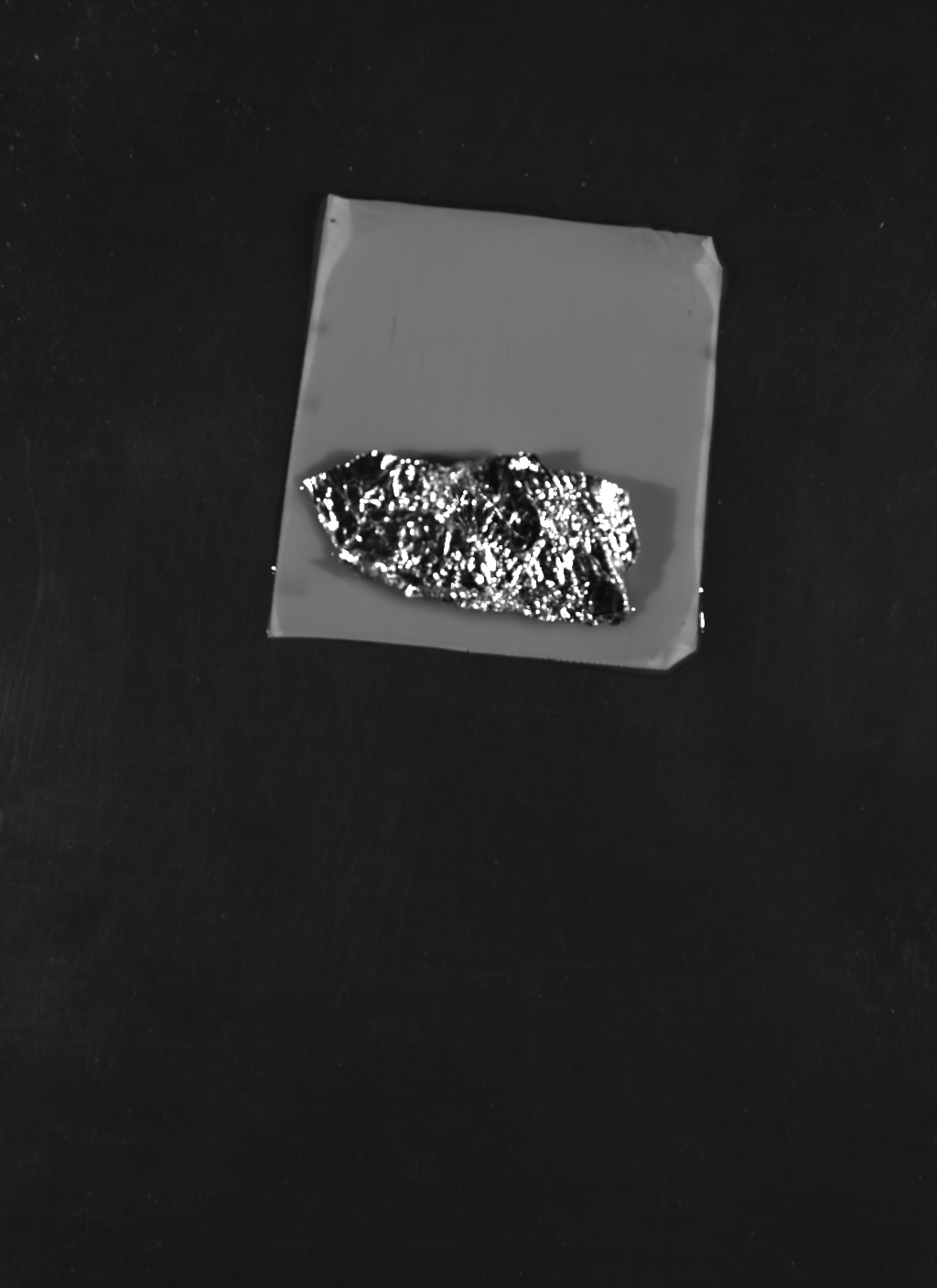

Supplement: Supplementary file 2 [file DataSheet_2.zip › wb Fig 5 C E/dyx-0514-B7H3-1 2021.05.13_16.16.53_Ch/dyx-0514-1-B7H3-1 2021.05.13_16.16.53_Ch-Marker.tif]

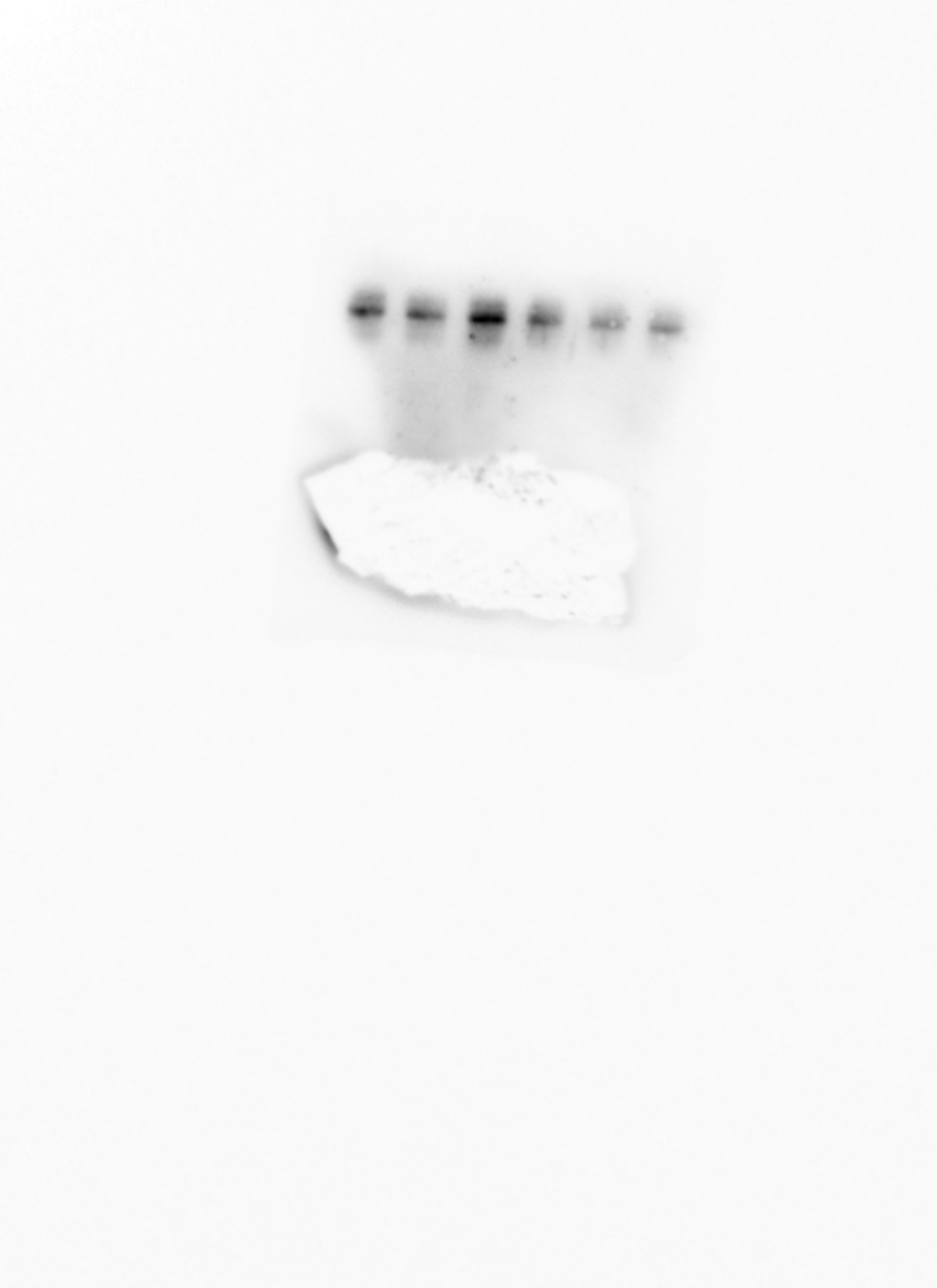

Supplement: Supplementary file 2 [file DataSheet_2.zip › wb Fig 5 C E/dyx-0514-B7H3-1 2021.05.13_16.16.53_Ch/dyx-0514-1-B7H3-1 2021.05.13_16.16.53_Ch.tif]

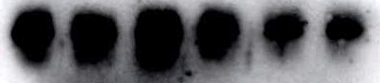

Supplement: Supplementary file 2 [file DataSheet_2.zip › wb Fig 5 C E/dyx-0514-B7H3-1 2021.05.13_16.16.53_Ch/F B7H3.tif]

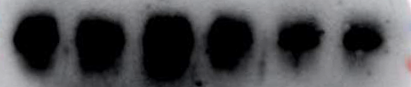

Supplement: Supplementary file 2 [file DataSheet_2.zip › wb Fig 5 C E/dyx-0514-B7H3-1 2021.05.13_16.16.53_Ch/psdyx-0514-1-B7H3-1 2021.05.13_16.16.53_Ch+Marker.tif]

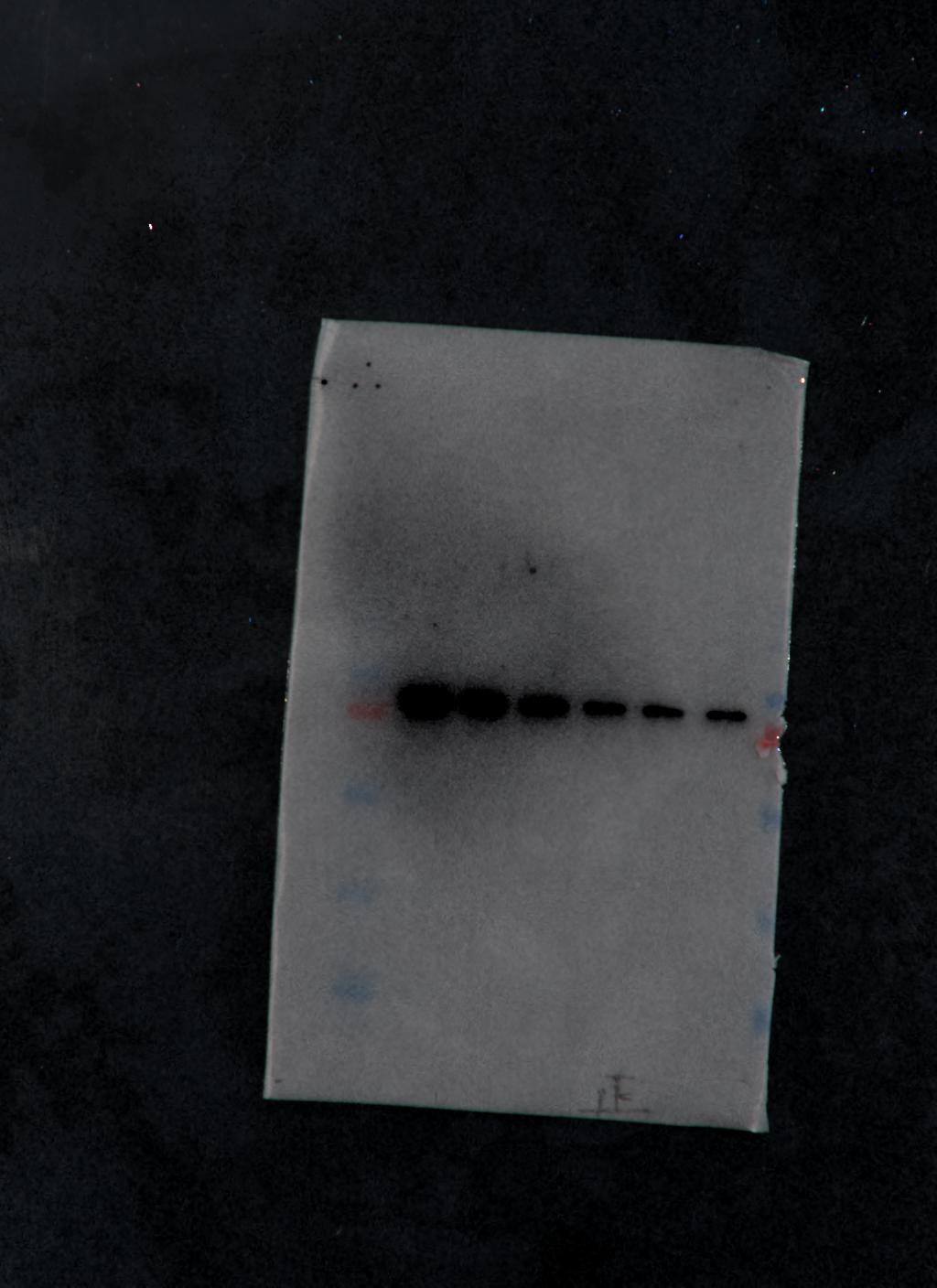

Supplement: Supplementary file 2 [file DataSheet_2.zip › wb Fig 5 C E/dyx-0517-stat1-3 2021.05.17_19.41.43_Ch/dyx-0517-3-stat1-3 2021.05.17_19.41.43_Ch+Marker.jpg]

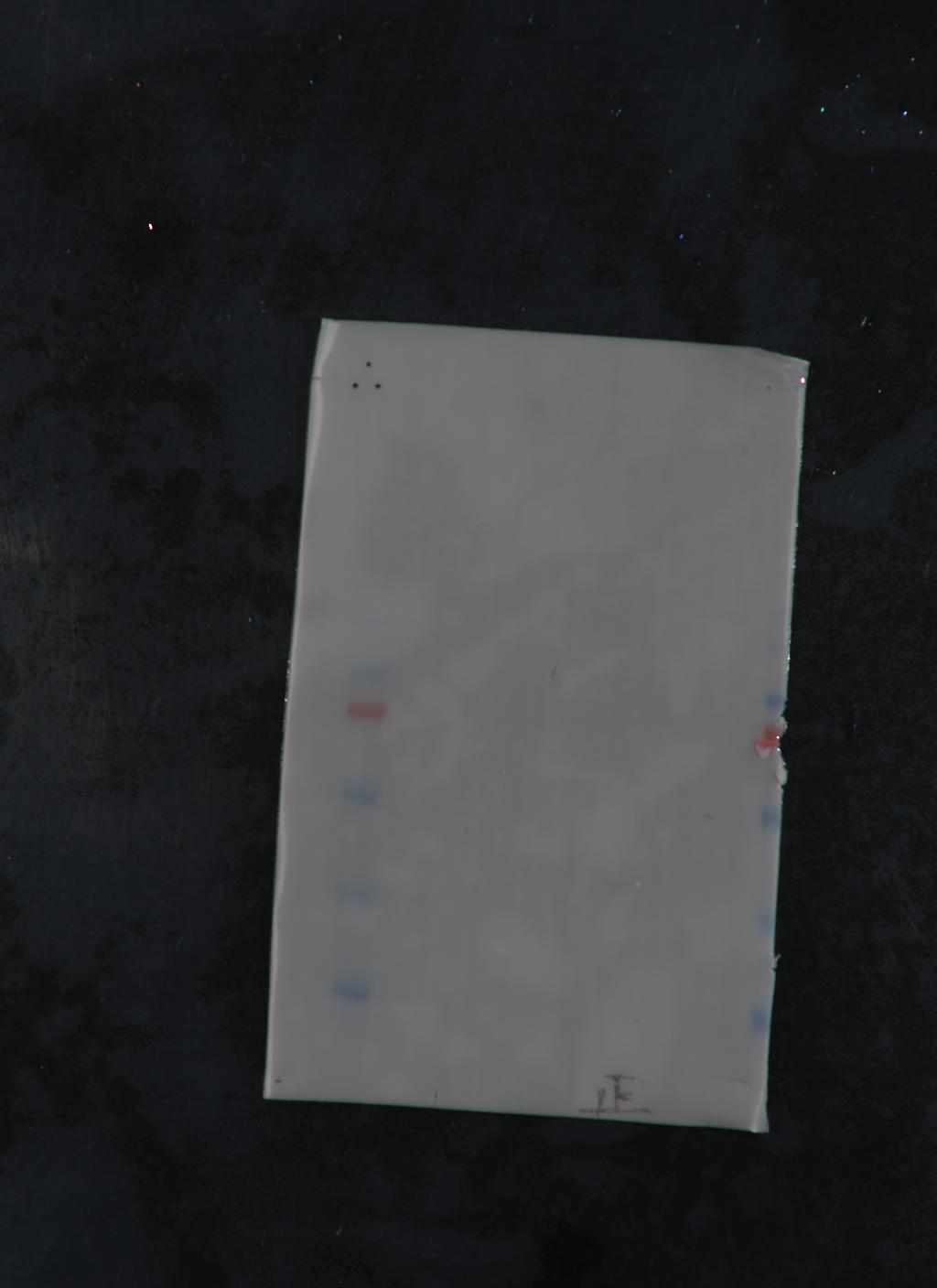

Supplement: Supplementary file 2 [file DataSheet_2.zip › wb Fig 5 C E/dyx-0517-stat1-3 2021.05.17_19.41.43_Ch/dyx-0517-3-stat1-3 2021.05.17_19.41.43_Ch-Marker.jpg]

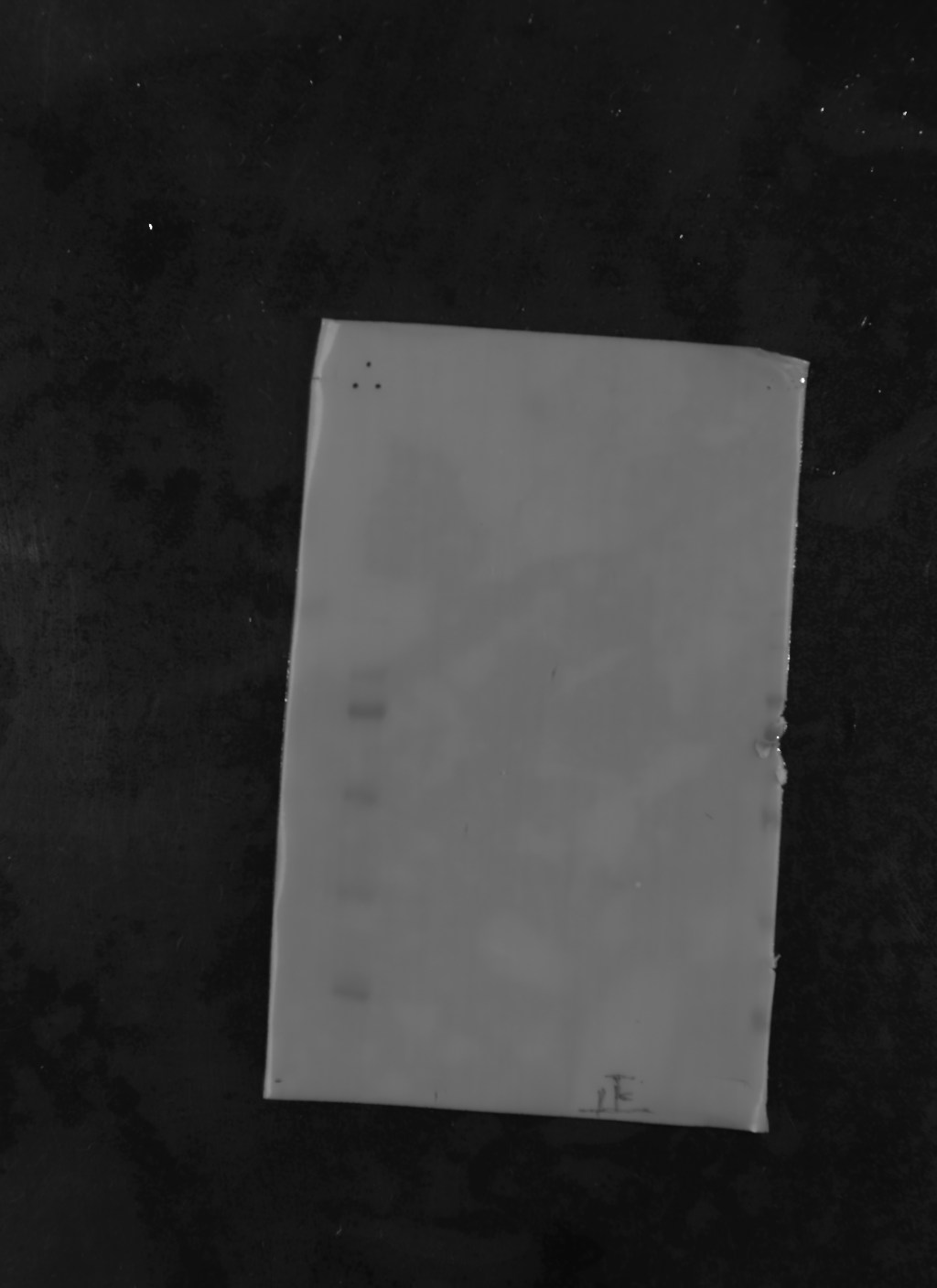

Supplement: Supplementary file 2 [file DataSheet_2.zip › wb Fig 5 C E/dyx-0517-stat1-3 2021.05.17_19.41.43_Ch/dyx-0517-3-stat1-3 2021.05.17_19.41.43_Ch-Marker.tif]

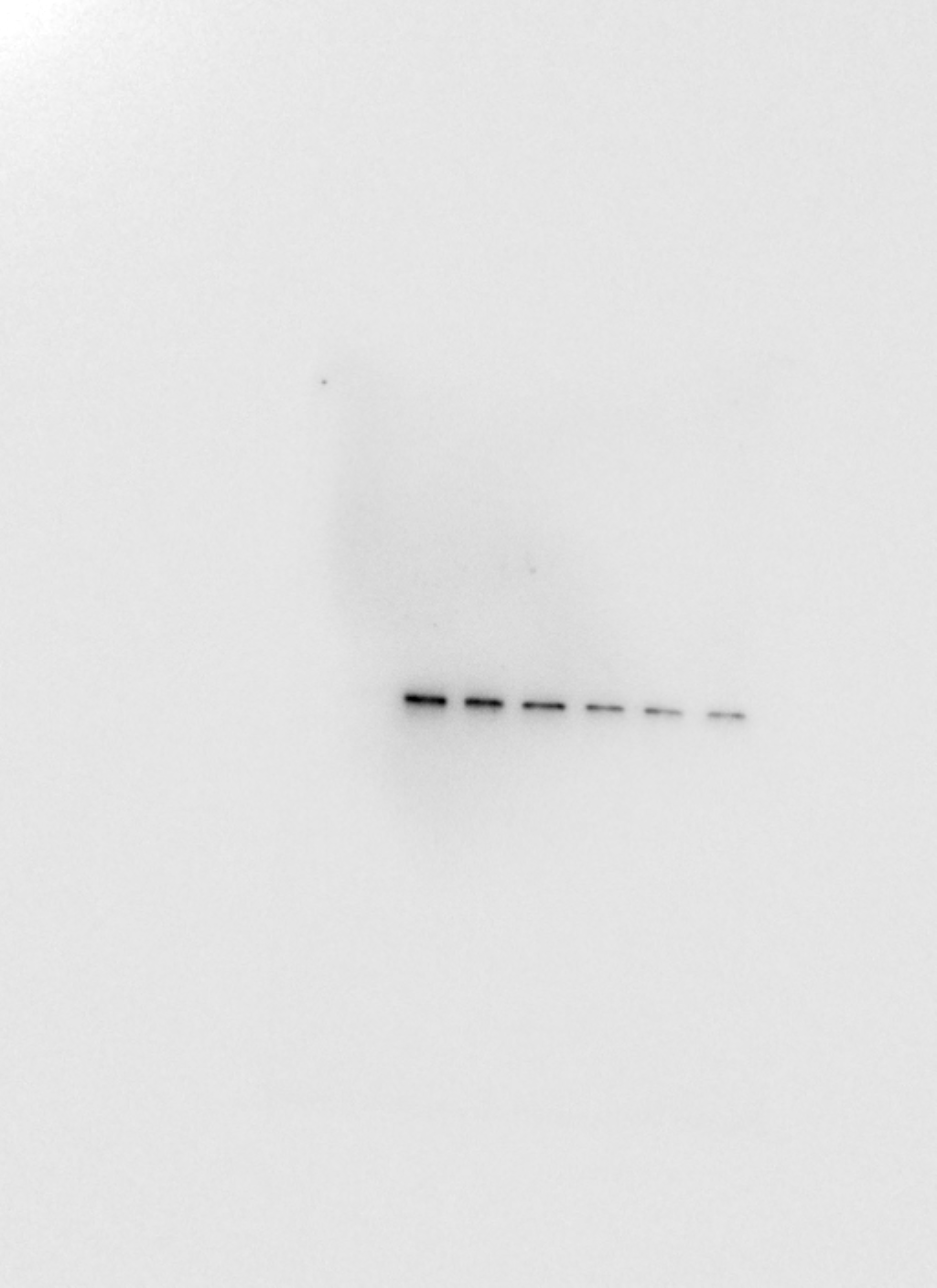

Supplement: Supplementary file 2 [file DataSheet_2.zip › wb Fig 5 C E/dyx-0517-stat1-3 2021.05.17_19.41.43_Ch/dyx-0517-3-stat1-3 2021.05.17_19.41.43_Ch.tif]

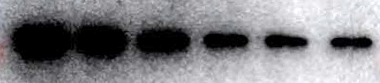

Supplement: Supplementary file 2 [file DataSheet_2.zip › wb Fig 5 C E/dyx-0517-stat1-3 2021.05.17_19.41.43_Ch/F STAT1.tif]

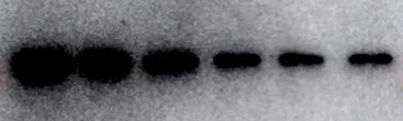

Supplement: Supplementary file 2 [file DataSheet_2.zip › wb Fig 5 C E/dyx-0517-stat1-3 2021.05.17_19.41.43_Ch/psdyx-0517-3-stat1-3 2021.05.17_19.41.43_Ch+Marker.tif]

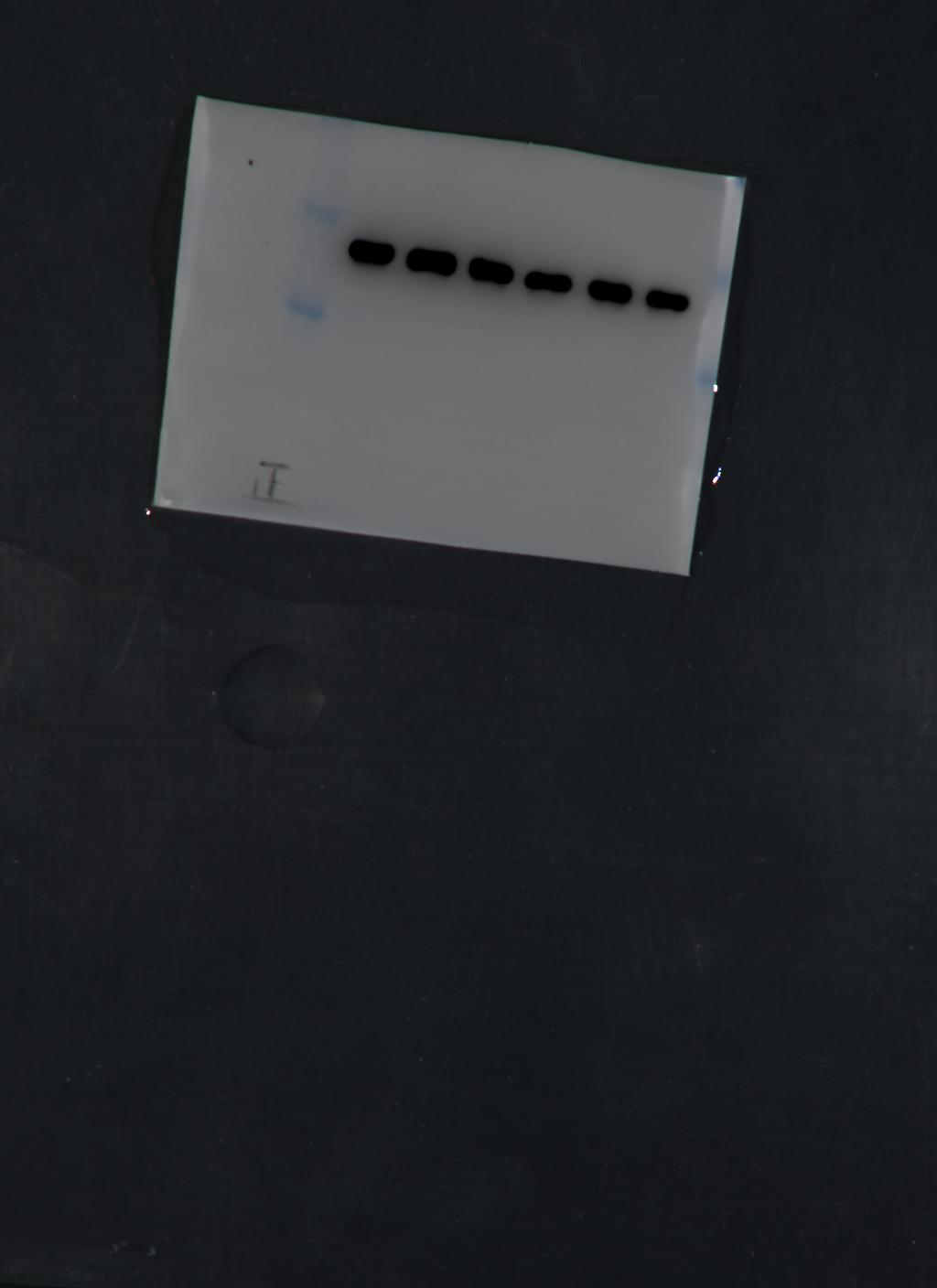

Supplement: Supplementary file 2 [file DataSheet_2.zip › wb Fig 5 C E/dyx-520-actin-1 2021.05.21_01.04.50_Ch/dyx-520-1-actin-1 2021.05.21_01.04.50_Ch+Marker.jpg]

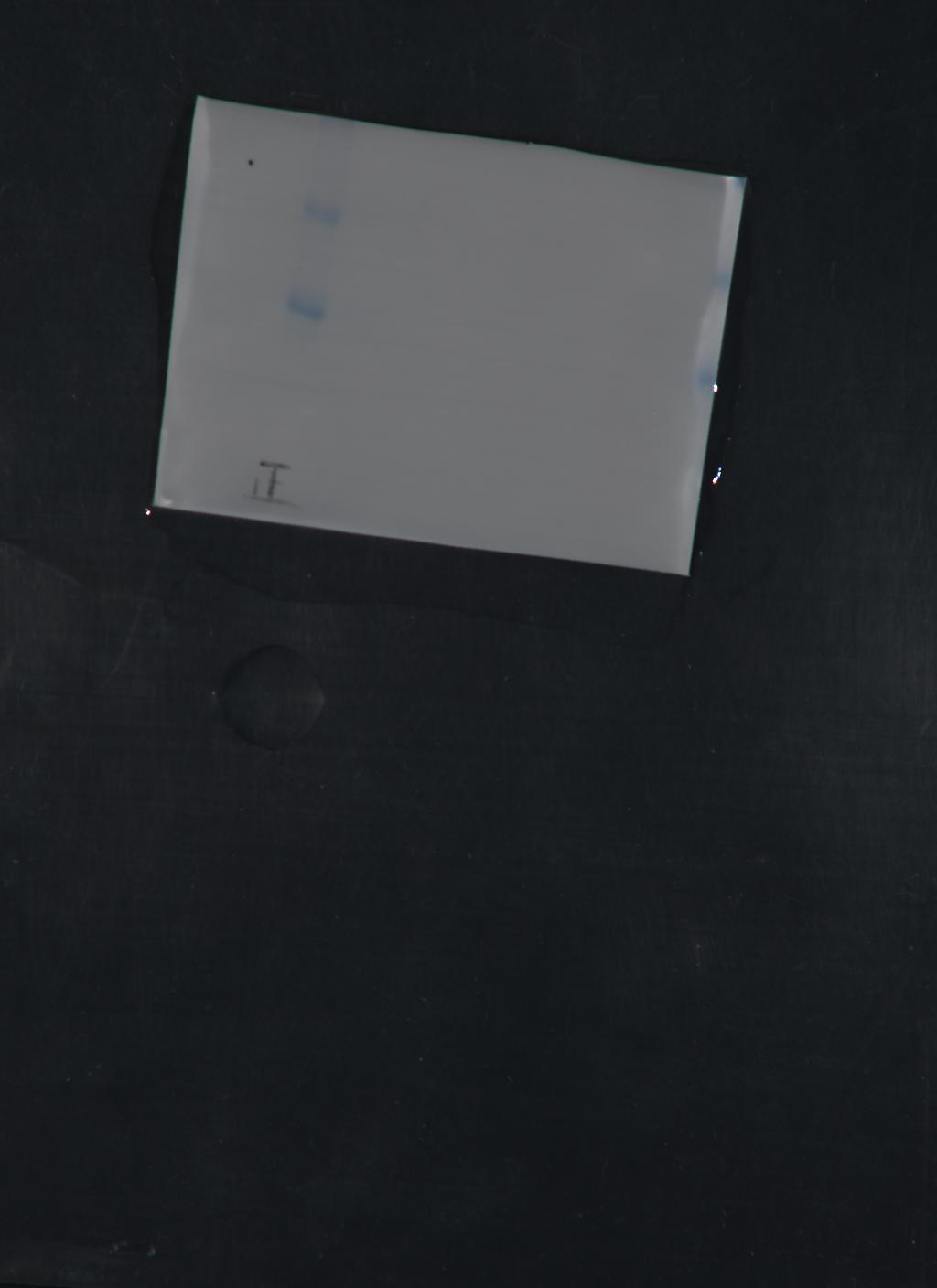

Supplement: Supplementary file 2 [file DataSheet_2.zip › wb Fig 5 C E/dyx-520-actin-1 2021.05.21_01.04.50_Ch/dyx-520-1-actin-1 2021.05.21_01.04.50_Ch-Marker.jpg]

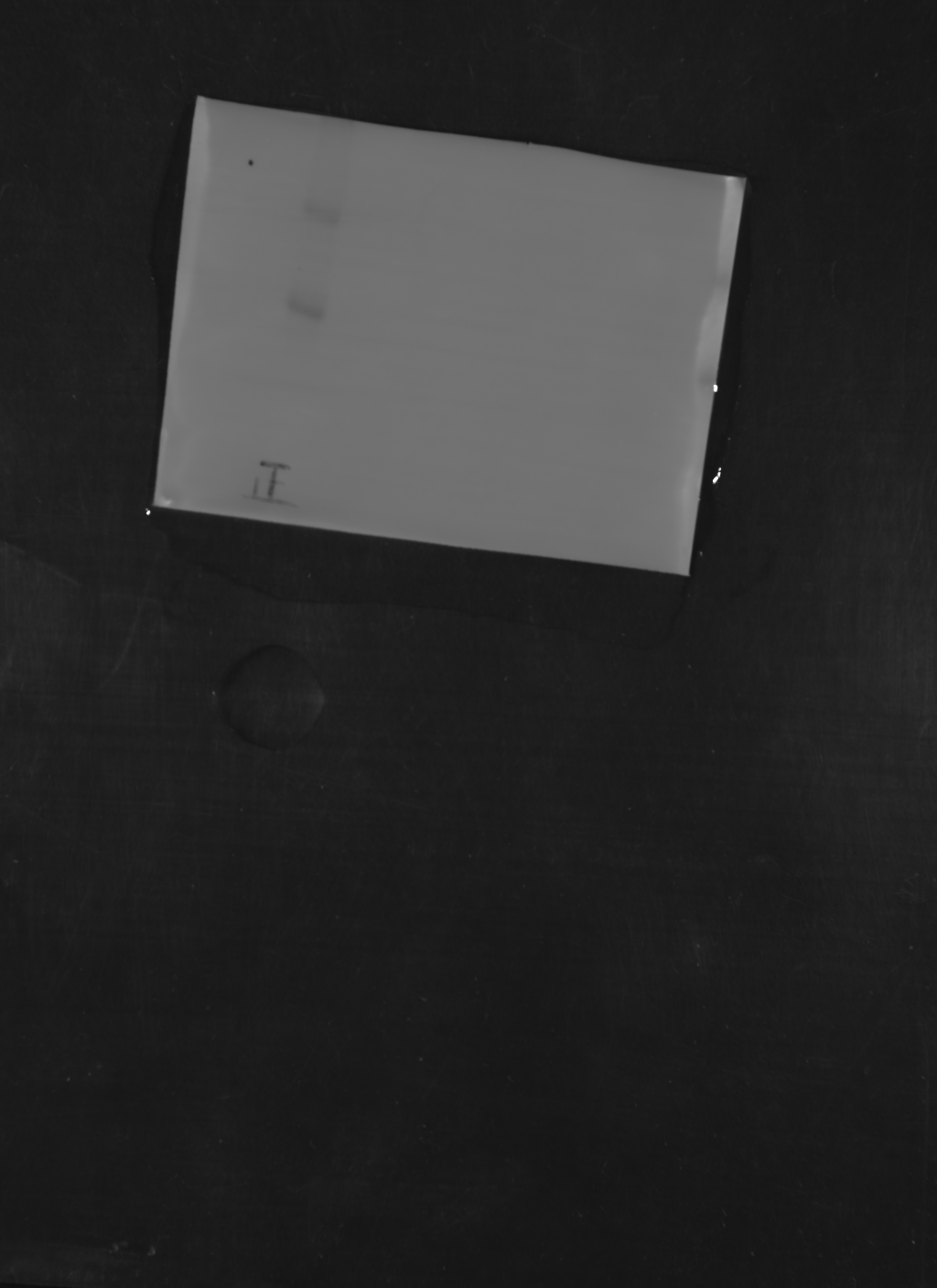

Supplement: Supplementary file 2 [file DataSheet_2.zip › wb Fig 5 C E/dyx-520-actin-1 2021.05.21_01.04.50_Ch/dyx-520-1-actin-1 2021.05.21_01.04.50_Ch-Marker.tif]

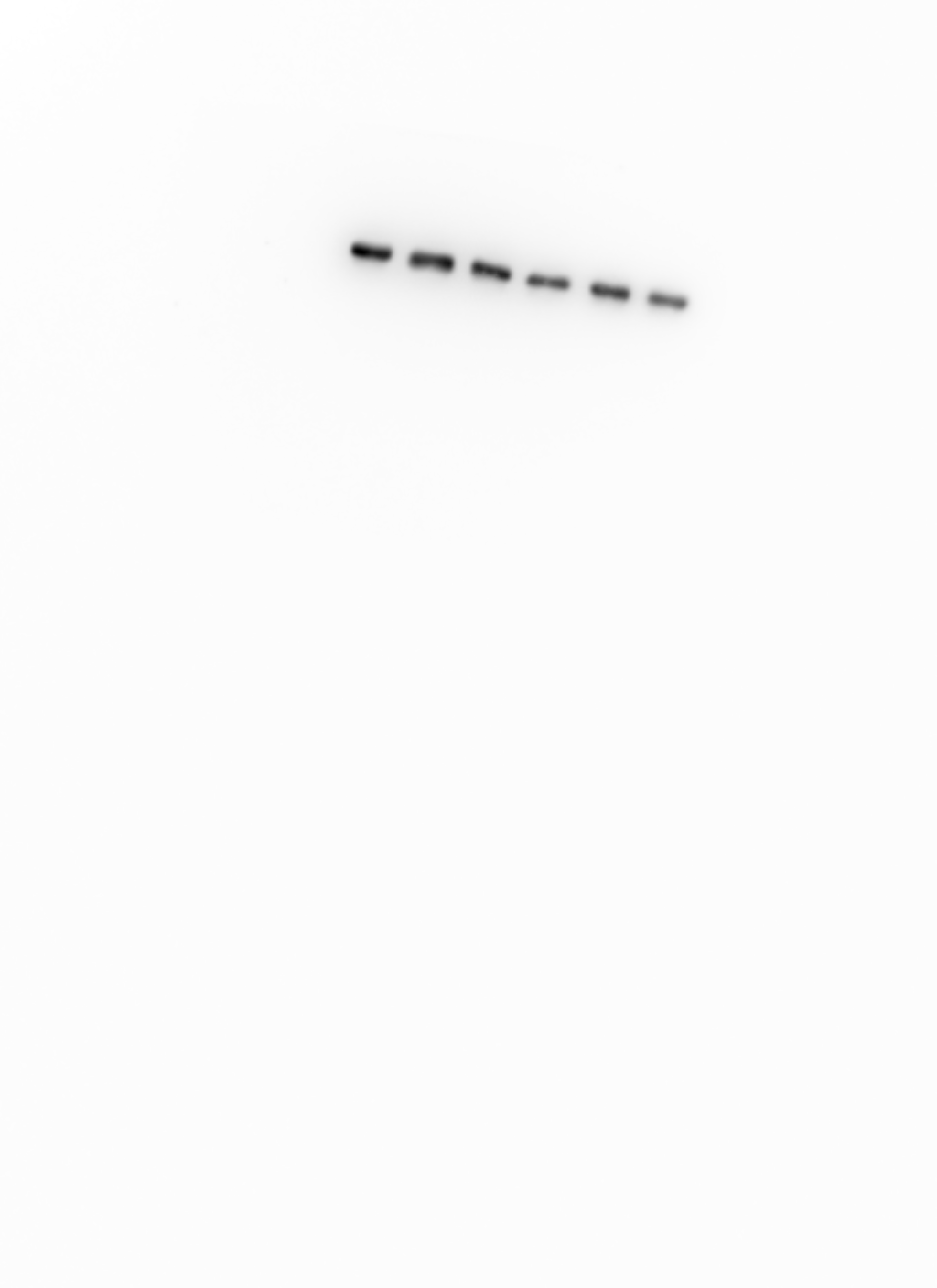

Supplement: Supplementary file 2 [file DataSheet_2.zip › wb Fig 5 C E/dyx-520-actin-1 2021.05.21_01.04.50_Ch/dyx-520-1-actin-1 2021.05.21_01.04.50_Ch.tif]

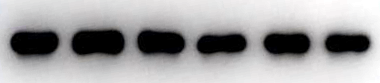

Supplement: Supplementary file 2 [file DataSheet_2.zip › wb Fig 5 C E/dyx-520-actin-1 2021.05.21_01.04.50_Ch/F actin.tif]

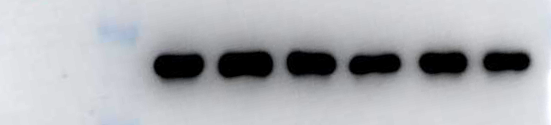

Supplement: Supplementary file 2 [file DataSheet_2.zip › wb Fig 5 C E/dyx-520-actin-1 2021.05.21_01.04.50_Ch/psdyx-520-1-actin-1 2021.05.21_01.04.50_Ch+Marker.tif]

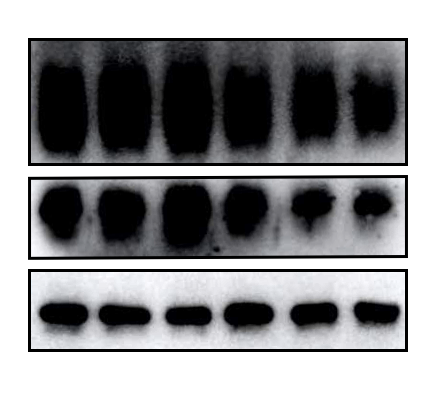

Supplement: Supplementary file 2 [file DataSheet_2.zip › wb Fig 5 C E/PD-L1.tif]

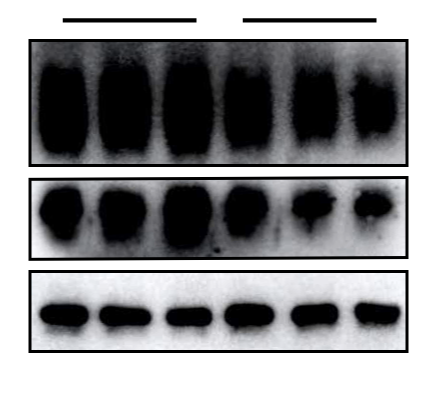

Supplement: Supplementary file 2 [file DataSheet_2.zip › wb Fig 5 C E/PD-L1终.tif]

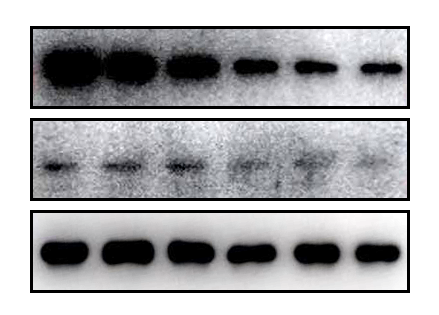

Supplement: Supplementary file 2 [file DataSheet_2.zip › wb Fig 5 C E/stat1.tif]

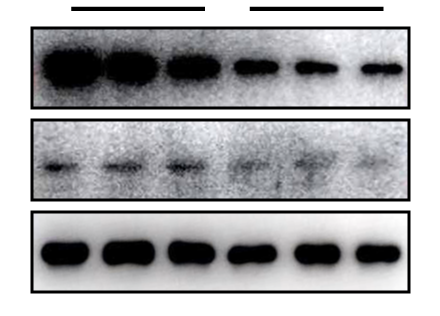

Supplement: Supplementary file 2 [file DataSheet_2.zip › wb Fig 5 C E/stat1终.tif]

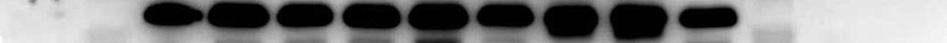

Supplement: Supplementary file 3 [file DataSheet_3.zip › wb Fig 3 B/dyx-0207-2-GAPDH-2 2021.02.07_19.03.40_Ch/dyx-0207-2-GAPDH-2 2021.02.07_19.03.40ps_Ch+Marker.jpg]

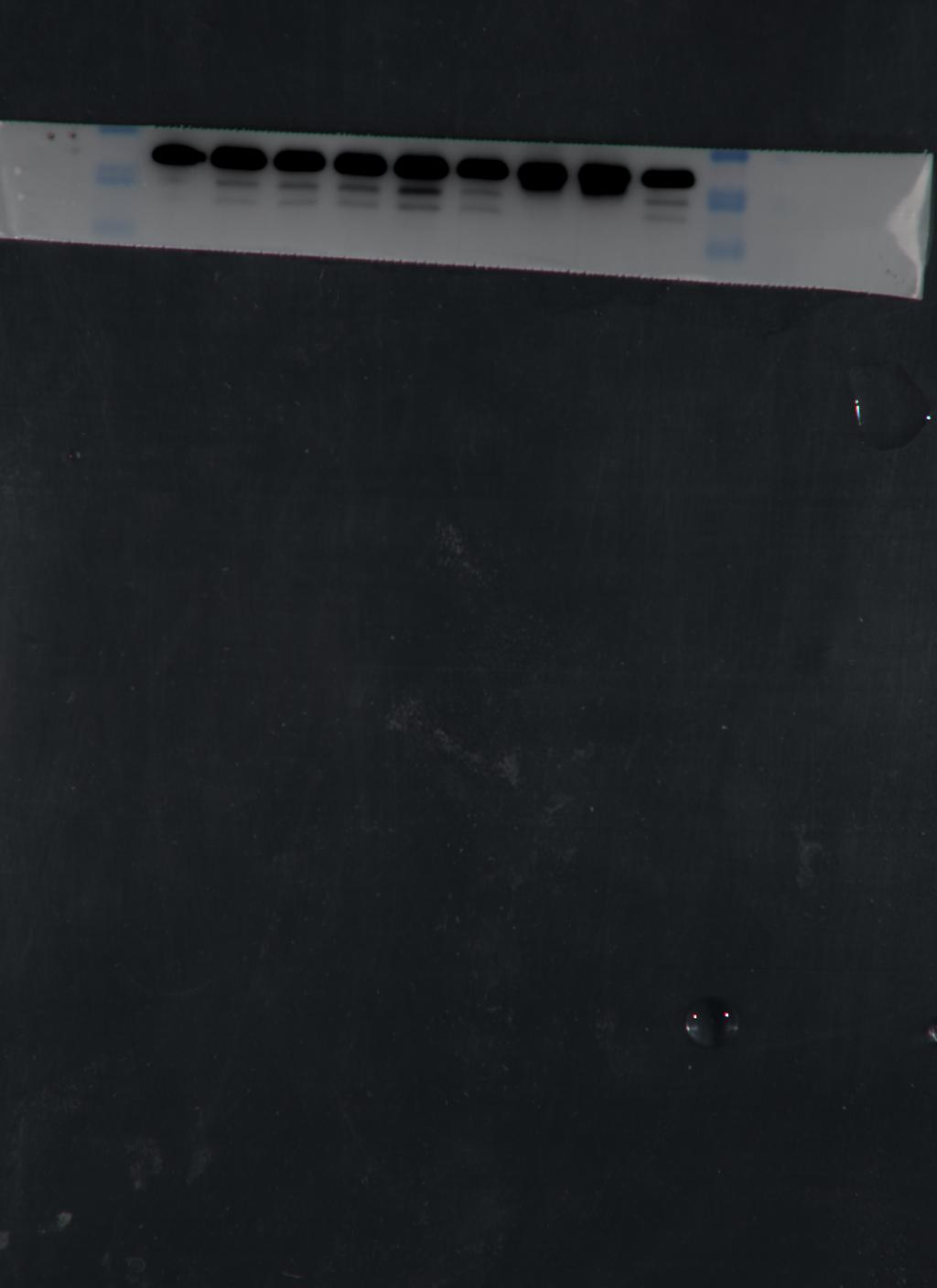

Supplement: Supplementary file 3 [file DataSheet_3.zip › wb Fig 3 B/dyx-0207-2-GAPDH-2 2021.02.07_19.03.40_Ch/dyx-0207-2-GAPDH-2 2021.02.07_19.03.40_Ch+Marker.jpg]

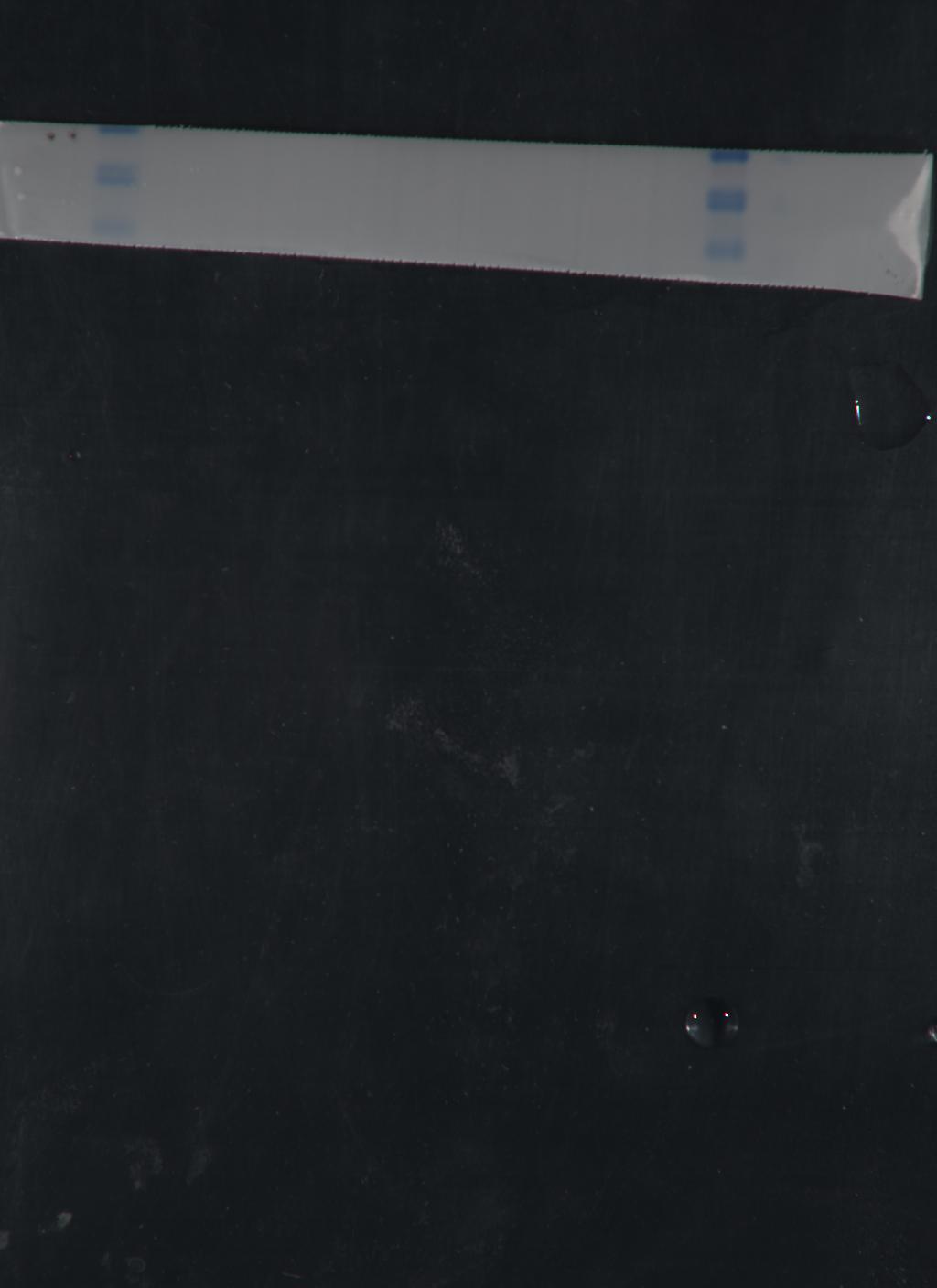

Supplement: Supplementary file 3 [file DataSheet_3.zip › wb Fig 3 B/dyx-0207-2-GAPDH-2 2021.02.07_19.03.40_Ch/dyx-0207-2-GAPDH-2 2021.02.07_19.03.40_Ch-Marker.jpg]

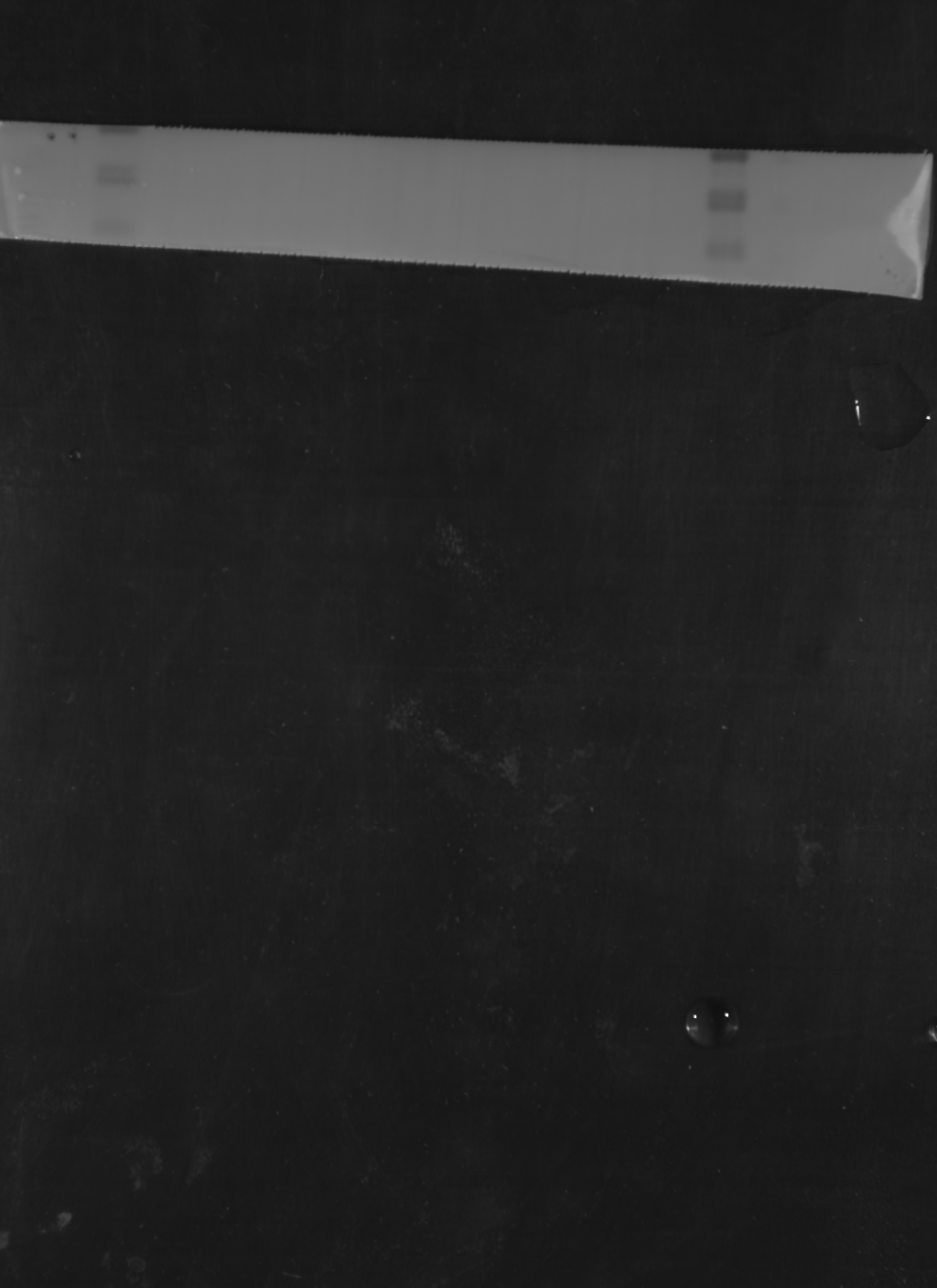

Supplement: Supplementary file 3 [file DataSheet_3.zip › wb Fig 3 B/dyx-0207-2-GAPDH-2 2021.02.07_19.03.40_Ch/dyx-0207-2-GAPDH-2 2021.02.07_19.03.40_Ch-Marker.tif]

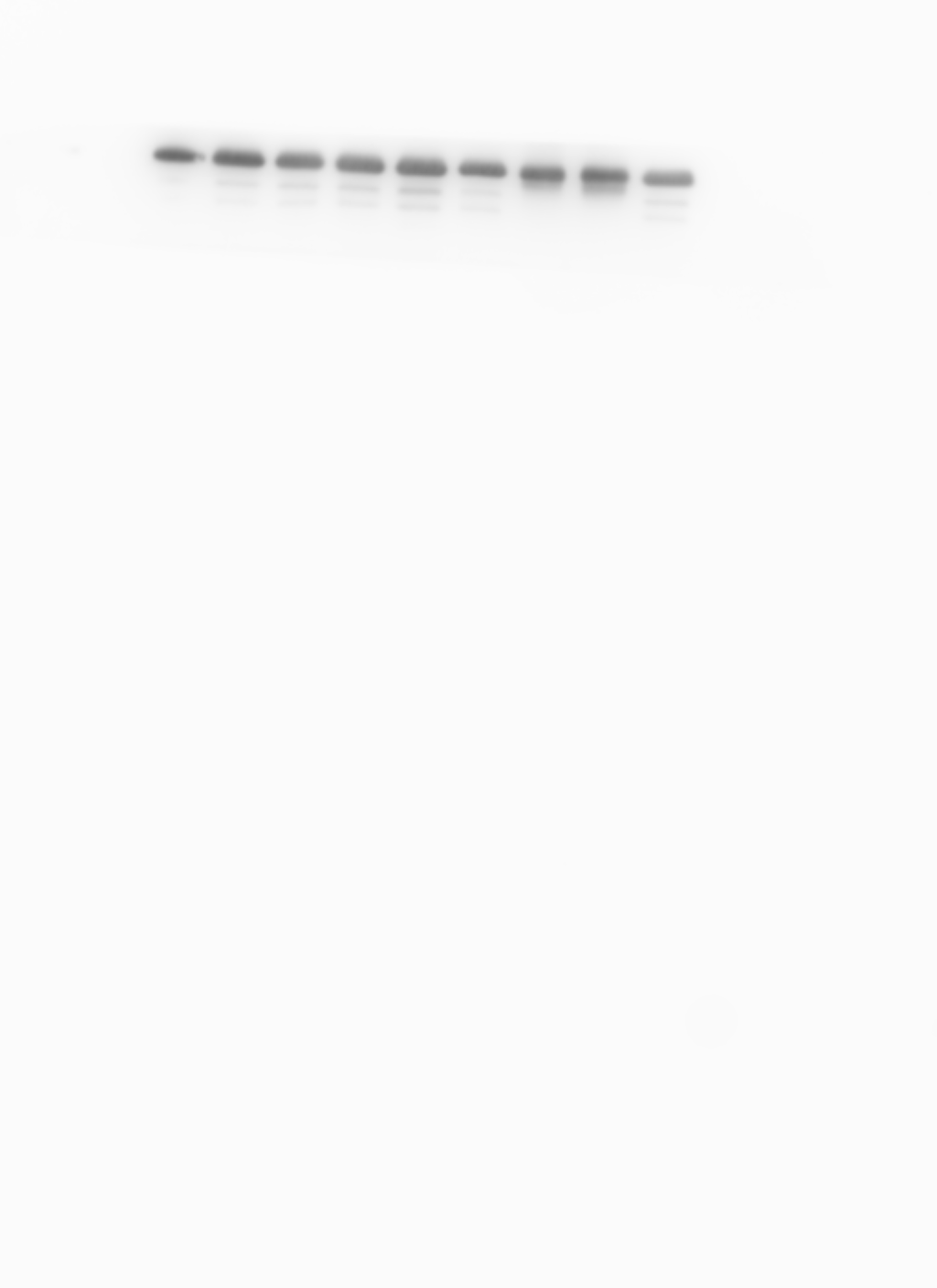

Supplement: Supplementary file 3 [file DataSheet_3.zip › wb Fig 3 B/dyx-0207-2-GAPDH-2 2021.02.07_19.03.40_Ch/dyx-0207-2-GAPDH-2 2021.02.07_19.03.40_Ch.tif]

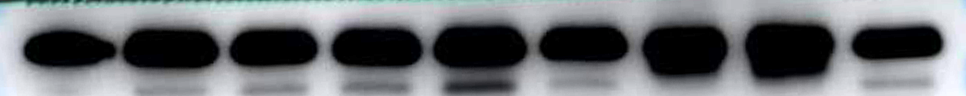

Supplement: Supplementary file 3 [file DataSheet_3.zip › wb Fig 3 B/dyx-0207-2-GAPDH-2 2021.02.07_19.03.40_Ch/F GAPDH.tif]

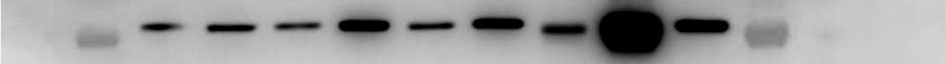

Supplement: Supplementary file 3 [file DataSheet_3.zip › wb Fig 3 B/dyx-0207-2-NAMPT-2 2021.02.07_19.01.12_Ch/dyx-0207-2-NAMPT-2 2021.02.07ps_19.01.12_Ch+Marker.jpg]

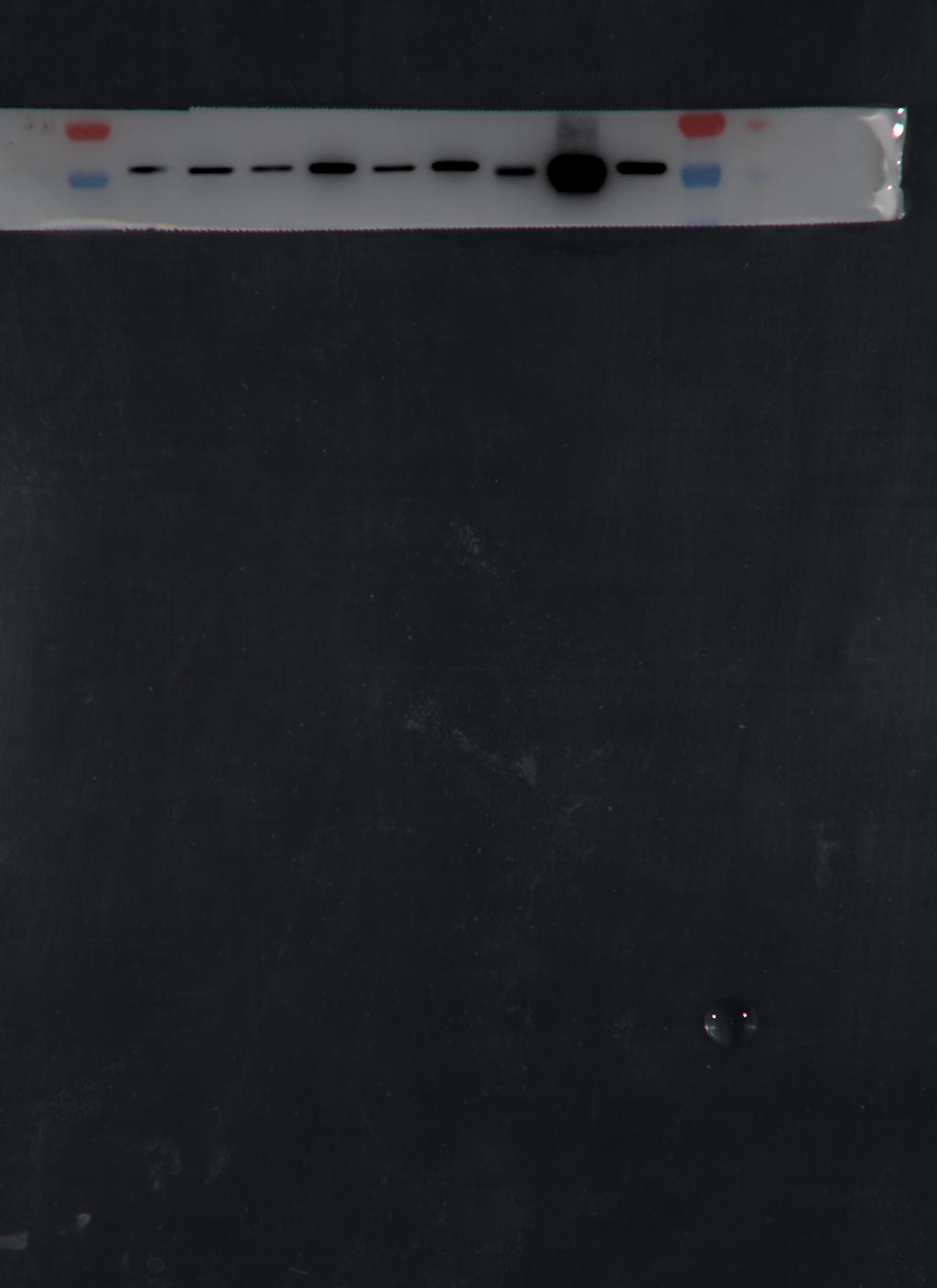

Supplement: Supplementary file 3 [file DataSheet_3.zip › wb Fig 3 B/dyx-0207-2-NAMPT-2 2021.02.07_19.01.12_Ch/dyx-0207-2-NAMPT-2 2021.02.07_19.01.12_Ch+Marker.jpg]

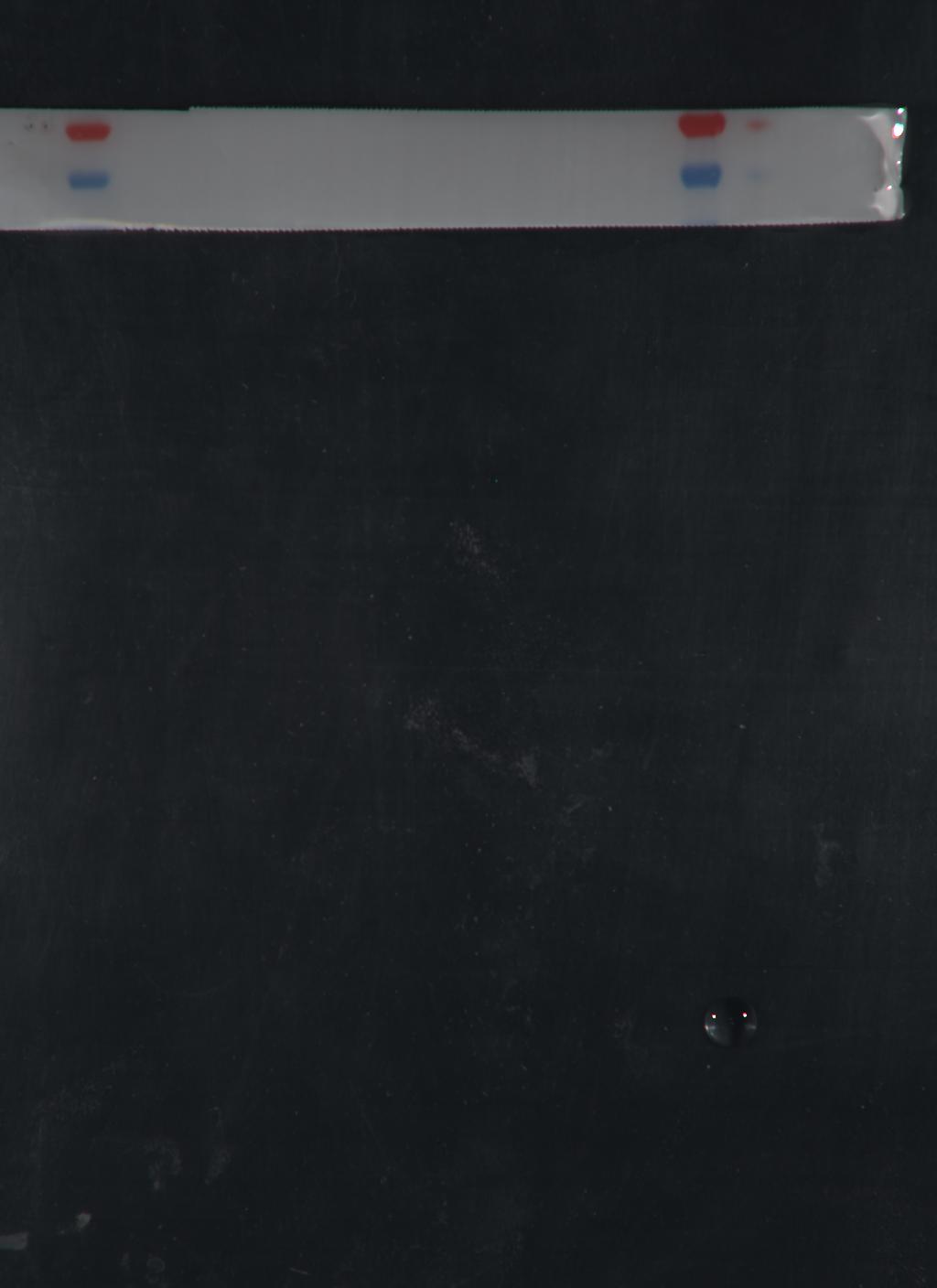

Supplement: Supplementary file 3 [file DataSheet_3.zip › wb Fig 3 B/dyx-0207-2-NAMPT-2 2021.02.07_19.01.12_Ch/dyx-0207-2-NAMPT-2 2021.02.07_19.01.12_Ch-Marker.jpg]

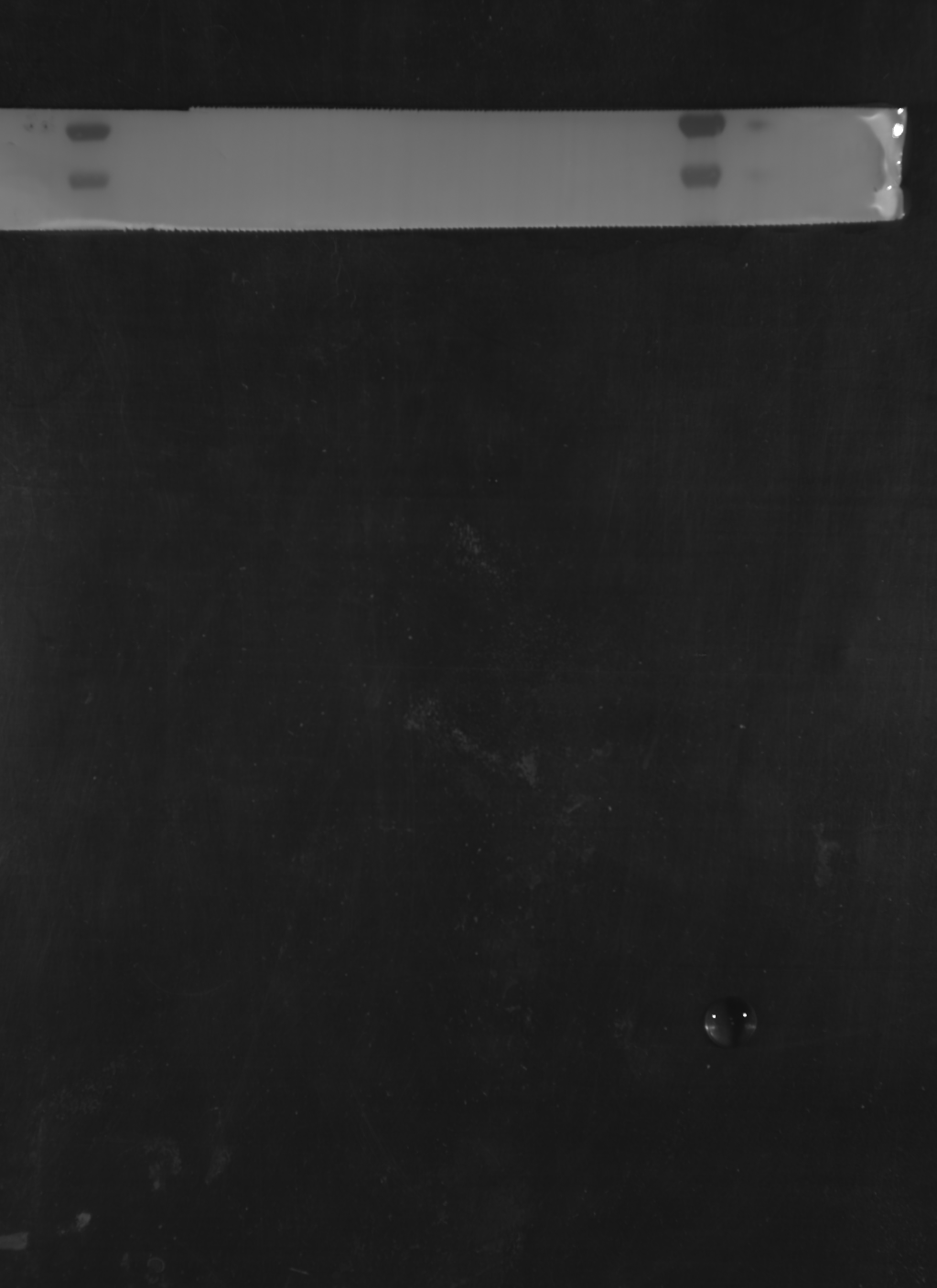

Supplement: Supplementary file 3 [file DataSheet_3.zip › wb Fig 3 B/dyx-0207-2-NAMPT-2 2021.02.07_19.01.12_Ch/dyx-0207-2-NAMPT-2 2021.02.07_19.01.12_Ch-Marker.tif]

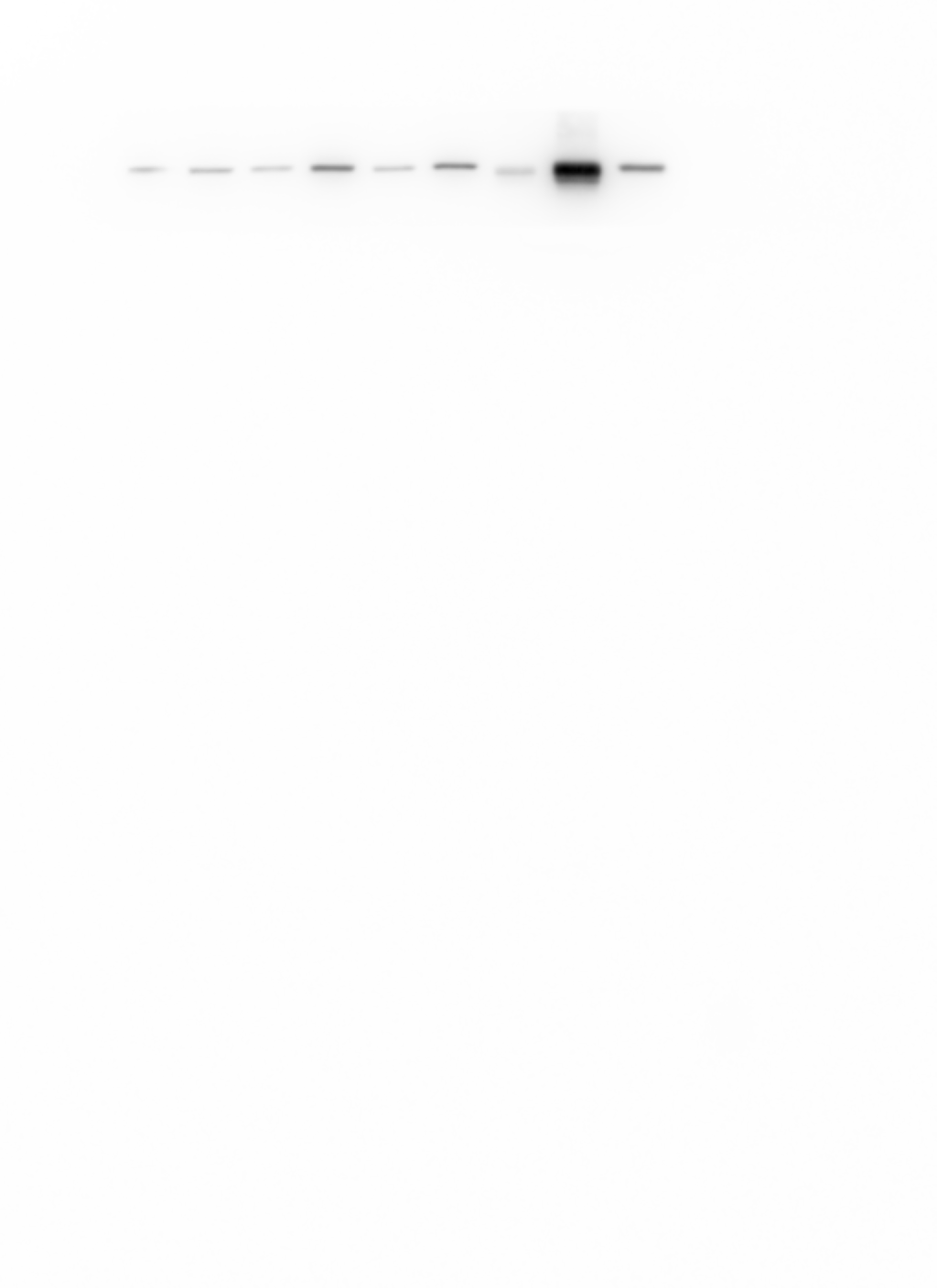

Supplement: Supplementary file 3 [file DataSheet_3.zip › wb Fig 3 B/dyx-0207-2-NAMPT-2 2021.02.07_19.01.12_Ch/dyx-0207-2-NAMPT-2 2021.02.07_19.01.12_Ch.tif]

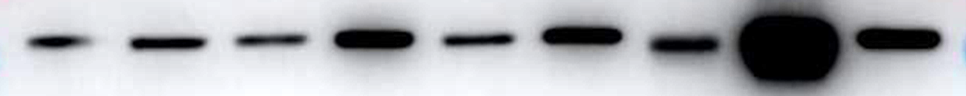

Supplement: Supplementary file 3 [file DataSheet_3.zip › wb Fig 3 B/dyx-0207-2-NAMPT-2 2021.02.07_19.01.12_Ch/F NAMPT.tif]
